# A novel protein encoded by ZCRB1-induced circHEATR5B suppresses aerobic glycolysis of GBM through phosphorylation of JMJD5

**DOI:** 10.1186/s13046-022-02374-6

**Published:** 2022-05-10

**Authors:** Jian Song, Jian Zheng, Xiaobai Liu, Weiwei Dong, Chunqing Yang, Di Wang, Xuelei Ruan, Yubo Zhao, Libo Liu, Ping Wang, Mengyang Zhang, Yunhui Liu

**Affiliations:** 1 Department of Neurosurgery, Shengjing Hospital of China Medical University, Shenyang, 110004 China; 2 Key Laboratory of Neuro-oncology in Liaoning Province, Shenyang, 110004 China; 3 Liaoning Medical Surgery and Rehabilitation Robot Technology Engineering Research Center, Shenyang, 110004 China; 4 Department of Neurobiology, College of Basic Medicine, China Medical University, Shenyang, 110122 China

**Keywords:** Glioblastoma multiforme, Aerobic glycolysis, ZCRB1, circHEATR5B, JMJD5, PKM2, Phosphorylation

## Abstract

**Background:**

RNA-binding proteins (RBPs) and circular RNAs (circRNAs) play important roles in glioblastoma multiforme (GBM). Aerobic glycolysis is a metabolic characteristic of GBM. However, the roles of RBPs and circRNAs in aerobic glycolysis in GBM remain unclear. The aim of this study is to explore the mechanisms by which RBPs and circRNAs regulate aerobic glycolysis in GBM cells.

**Methods:**

RNA sequencing and circRNA microarray analysis were performed to identify RBPs and circRNAs for further study. Mass spectrometry validated the encoded protein and its interacting proteins. Quantitative reverse transcription PCR and western blot assays were used to determine the mRNA and protein expression, respectively. Furthermore, immunofluorescence and fluorescence in situ hybridization assays were used to determine the protein and RNA localization, respectively. Glucose and lactate measurement assays, Seahorse XF glycolysis stress assays and cell viability assays were conducted to investigate the effects on glycolysis and proliferation in GBM cells.

**Results:**

We selected zinc finger CCHC-type and RNA-binding motif 1 (ZCRB1) and circRNA HEAT repeat containing 5B (circHEATR5B) as candidates for this study. These genes were expressed at low levels in GBM tissues and cells. Both ZCRB1 and circHEATR5B overexpression suppressed aerobic glycolysis and proliferation in GBM cells. ZCRB1 overexpression promoted the Alu element-mediated formation of circHEATR5B. In addition, circHEATR5B encoded a novel protein HEATR5B-881aa which interacted directly with Jumonji C-domain-containing 5 (JMJD5) and reduced its stability by phosphorylating S361. JMJD5 knockdown increased pyruvate kinase M2 (PKM2) enzymatic activity and suppressed glycolysis and proliferation in GBM cells. Finally, ZCRB1, circHEATR5B and HEATR5B-881aa overexpression inhibited GBM xenograft growth and prolonged the survival time of nude mice.

**Conclusions:**

This study reveals a novel mechanism of regulating aerobic glycolysis and proliferation in GBM cells through the ZCRB1/circHEATR5B/HEATR5B-881aa/JMJD5/PKM2 pathway, which can provide novel strategies and potential targets for GBM therapy.

**Supplementary Information:**

The online version contains supplementary material available at 10.1186/s13046-022-02374-6.

## Background

Glioblastoma multiforme (GBM) is a primary malignant tumor in the central nervous system with high morbidity and mortality rates, characterized by aggressive growth and a high recurrence rate [1]. The most widely accepted therapeutic approach for GBM is surgery with radiotherapy plus temozolomide, followed by an adjuvant phase with temozolomide alone [2]. However, the current treatment protocol cannot markedly improve the poor prognosis of GBM patients, [3] with a median survival of only 15 months [4]. Energy metabolism reprogramming has been identified as one of ten hallmarks of cancer, [5] so anti-metabolic therapy has become a new research direction for GBM treatment.

Most tumor cells prefer to obtain energy through glycolysis rather than oxidative phosphorylation even in the presence of sufficient oxygen, a phenomenon known as aerobic glycolysis or the Warburg effect, [6] as it is more efficient in producing energy and large amounts of intermediates for biosynthesis [7]. Aerobic glycolysis has been reported to promote the development of GBM cells [8]. Therefore, studies on aerobic glycolysis may provide novel targets for GBM therapy.

As one of the three rate-limiting enzymes of glycolysis, pyruvate kinase includes four isozymes, of which M2-type pyruvate kinase (PKM2) is mainly expressed in rapidly proliferating cells, especially tumor cells, and it plays a crucial role in the development of tumors, such as GBM, [9] by regulating aerobic glycolysis. PKM2 mainly exists as a high-activity tetramer in normal proliferating cells or a low-activity dimer in tumor cells [10]. In the cytoplasm of tumor cells, low-activity PKM2 can prompt the lactate fermentation of pyruvate [11] and the accumulation of intermediate metabolites for biosynthesis [12]. In addition, several studies have shown that the PKM2 dimer can translocate into the nucleus of tumor cells through various mechanisms, and act as a protein kinase or transcription regulator to promote aerobic glycolysis [13].

RNA-binding proteins (RBPs) are a class of proteins that play a posttranscriptional regulatory role in tumor cells by binding to RNA. For example, RBP-Lin28A promotes proliferation, migration and invasion, and inhibits apoptosis in osteosarcoma cells by increasing the stability of lncRNA-MALAT1 [14]. Zinc finger CCHC-type and RNA-binding motif 1 (ZCRB1) is an RBP encoded by the human *ZCRB1* gene, mapped to chromosome 12q12. Researchers have demonstrated that ZCRB1 can bind specifically to the 5′-UTR of SARS-CoV RNA and promote viral RNA synthesis [15]. However, the expression and functions of ZCRB1 in GBM cells have not been investigated.

Circular RNAs (circRNAs) are a class of noncoding RNAs whose 3′ and 5′ ends are covalently bound via back-splicing, [16] which can exert important regulatory effects on tumorigenesis and tumor development as microRNA sponges and protein scaffolds [17, 18]. Recent studies have indicated that circRNAs can be translated, [19, 20] and circRNA-encoded proteins are reported to be functional in the progression of gastric and colon cancers [21, 22]. CircRNA HEAT repeat containing 5B (circHEATR5B), also named circ_0054048, is located in chr2:37,227,728-37,268,435 and consists of 15 exons with a length of 2849 nt. The circRNADb database [23] search suggested that circHEATR5B might encode a protein containing 881 amino acids (termed HEATR5B-881aa). However, the expression and functions of circHEATR5B and HEATR5B-881aa in GBM tissues and cells have not been reported.

Jumonji C-domain-containing 5 (JMJD5), also called lysine demethylase 8 (KDM8), has been reported to interact directly with PKM2, inducing PKM2 dimerization to regulate glucose metabolism reprogramming [24, 25]. Studies have noted that high expression of JMJD5 promotes migration and invasion, and inhibits apoptosis in oral squamous cell carcinoma [26]. JMJD5 also promotes breast cancer cell proliferation [27]. Nevertheless, the expression pattern and regulatory roles of JMJD5 in GBM cells require further study.

In this study, we detected the endogenous expressions of ZCRB1, circHEATR5B, HEATR5B-881aa and JMJD5 in GBM tissues and cells, demonstrated their regulatory effects on aerobic glycolysis and proliferation in GBM cells, and further explored the possible mechanisms. This study revealed a novel mechanism of aerobic glycolysis in GBM and provided new strategies for GBM therapy.

## Methods

### Human glioma and normal tissues

To detect the endogenous expressions of ZCRB1, circHEATR5B, HEATR5B-881aa and JMJD5, human glioma tissues were collected and divided into two parts: low-grade glioma tissues (LGGTs, WHO Grade I-II, *n* = 12) and high-grade glioma tissues (HGGTs, WHO Grade III-IV, *n* = 12) based on the World Health Organization classification guidelines for pathological grades in 2016 [28]. Adjacent non-tumor brain tissues (NBTs, *n* = 12) served as the negative control.

### Cell culture

The human GBM cell lines (U87, U251, U373, and A172) and 293 T cells were purchased from the Shanghai Institutes for Biological Sciences Cell Resource Center and were cultured in DMEM (HyClone, Logan, UT) supplemented with 10% fetal bovine serum (TBD, Tianjin, China) according to standard protocols. Normal human astrocytes (NHAs) were purchased from ScienCell Research Laboratories and were cultured in astrocyte medium (ScienCell, Carlsbad, CA). All cells were cultured at 37 °C in a 5% CO_2_ humidified incubator.

### RNA sequencing (RNA-seq)

Total RNAs were extracted from three GBM and NBTs using TRIzol reagent (Life Invitrogen, Carlsbad, CA) and then treated using the RiboMinus Eukaryote Kit (Qiagen, Valencia, CA) to remove rRNAs before generating RNA-seq library. Next, the RNA-seq library was deep sequenced with the Illumina HiSeq 2000. RNA sequencing reads were aligned to the human reference genome by software STAR and RNA abundance was quantified using software RSEM.

### Quantitative reverse transcription PCR assay (qRT–PCR)

Total RNAs were extracted from tissues and cells using TRIzol reagent. RNA concentration and quality were measured using NanoDrop2000 Spectrophotometer. The expressions of circHEATR5B and the mRNAs of ZCRB1, HEATR5B, JMJD5 and β-actin were detected using the One Step TB Green® PrimeScript™ RT-PCR Kit (TaKaRa, Liaoning, China) on the ABI 7500 Fast Real-Time PCR system. The primers are given in Additional file 2: Table S1. The results were calculated using the 2^-ΔΔCt^ relative quantification method and β-actin served as an endogenous control.

### Western blot assay

Total proteins were extracted from tissues and cells using RIPA lysis buffer (Beyotime, Shanghai, China). Western blot assay was performed as previously described [29]. Protein samples were added into SDS-PAGE gels followed by electrophoresis and subsequently transferred to PVDF membranes. PVDF membranes were incubated with primary antibodies overnight at 4 °C after blocking for 2 h at room temperature. The next day, after washing by TTBS, PVDF membranes were incubated with the corresponding secondary antibodies at room temperature for 2 h. Proteins were identified using the BeyoECL Star Kit (Beyotime, Shanghai, China) and captured by MicroChemi chemiluminescent imaging system (DNR, Jerusalem, Israel). ImageJ software was used for analyzing the bands and β-actin was used as an endogenous control.

The primary antibodies are as follows: ZCRB1 (Proteintech Cat# 25629–1-AP), JMJD5 (ABclonal Cat# A11606), β-actin (Proteintech Cat# 20536–1-AP), GST tag (Proteintech Cat# 66001–2-Ig), and FLAG tag (Proteintech Cat# 20543–1-AP). HEATR5B-881aa and p-JMJD5-S361 antibodies were prepared by Beijing Huada Protein Innovation. The secondary antibodies are as follows: Goat anti-mouse IgG (Proteintech Cat# SA00001–1), Goat anti-rabbit IgG (Proteintech Cat# SA00001–2).

### Immunofluorescence assay (IF)

Cells seeded on glass slides were fixed in 4% paraformaldehyde for 30 min and permeabilized with 0.2% TritonX-100 for 20 min and then blocked with 5% BSA for 2 h at room temperature. Next, the cell slides were incubated with primary antibodies at 4 °C overnight. Then, the cell slides were washed with PBST three times and incubated with fluorescent-conjugated secondary antibodies, Goat anti-rabbit Alexa Fluor 488 or Goat anti-rabbit Alexa Fluor 647 (Beyotime, Shanghai, China), for 2 h at room temperature away from the light. Finally, the nuclei were counterstained with DAPI for 5 min. Fluorescence was visualized under laser confocal microscopy.

### Cell transfection

Cells were seeded into 24-well plates and transfected with plasmids using Lipofectamine3000 reagent (Life Technologies, Carlsbad, CA) based on the experimental groupings when cell confluence reached 50–70%. For each well, 0.75 μl Lipofectamine3000 reagent was diluted with 25 μl Opti-MEM Medium (Thermo Fisher, Waltham, MA). 500 ng plasmid DNA was diluted with 25 μl Opti-MEM Medium and 1 μl P3000 reagent. Then, the diluted DNA was incubated with diluted Lipofectamine3000 reagent for 10 min at room temperature. Finally, the mixture was added into the well and incubated with cells for 48 h at 37 °C. The stably transfected cells were screened by neomycin or puromycin and the transfection efficiency was detected by qRT–PCR and western blot assays.

The shRNAs against ZCRB1 (sh-ZCRB1: site #1, 5′-GCACAGTGTATGTATCCAA-3′; site #2, 5′-CTGACAAACAATGACTTGT-3′; site #3, 5′-GGGCAATAAACAACAAACA-3′) and the shRNAs against circHEATR5B (sh-circHEATR5B: site #1, 5′-CTCAACCAGGTTGAAATCGGC-3′; site #2, 5′-AACCAGGTTGAAATCGGCTCG-3′) were synthesized by GenePharma, the shRNAs against JMJD5 (sh-JMJD5: site #1, 5′-CCTGTTCATCCCGGTGAAATA-3′; site #2, 5′-GAGGAGGAAATCACCATCAAT-3′; site #3, 5′-GTCAACGAGTTCATCAGCAAA-3′) were synthesized by GeneChem, and their corresponding empty plasmids (sh-ZCRB1-NC, sh-circHEATR5B-NC, and sh-JMJD5-NC) were constructed as the negative control. The plasmids with ZCRB1 full-length sequence (OV-ZCRB1), circHEATR5B full-length sequence (OV-circHEATR5B) or mutant IRES (circHEATR5B-IRES-Mut), HEATR5B-881aa full-length sequence (OV-HEATR5B-881aa), JMJD5 full-length sequence (OV-JMJD5), JMJD5 with wild-type S361 (JMJD5-WT) or mutant S361 (JMJD5-S361A, JMJD5-S361E), and their corresponding empty plasmids were constructed by GenePharma.

### Cell viability assay

Cell viability was detected using the Cell Counting Kit-8 (Dojindo, Kumamoto, Japan) according to the manufacturer’s protocol. Briefly, cells (1 × 10^3^ in 100 μl) were seeded into 96-well plates and incubated at 37 °C for 48 h. Then, 10 μl of CCK-8 reagent was added into each well and incubated for 1–4 h at 37 °C. Finally, the absorbance at 450 nm was measured by SpectraMax M5 microplate reader.

### Glucose and lactate measurement assays

Glucose and lactate concentration were detected using the glucose assay kit and lactic acid assay kit (Nanjing Jiancheng, Jiangsu, China). In brief, cells (1 × 10^3^ per well) were seeded into 96-well plates with 100 μl medium. The supernatants of medium were collected after 24 h and added into glucose or lactate assay kit reagents according to the manufacturer’s protocol. The absorbance at corresponding wavelength was measured by SpectraMax M5 microplate reader. Finally, glucose consumption and lactate production were calculated.

### Seahorse XF glycolysis stress assay

Extracellular acidification rate (ECAR) was measured using the Seahorse XF Glycolysis Stress Test Kit (Agilent, Santa Clara, CA) and XF24 Extracellular Flux Analyzer, as previously described [30]. Briefly, cells (5 × 10^4^ per well) were seeded into Seahorse XF24 V7 PS Cell Culture Microplates and cultured overnight. ECAR was measured in XF base medium supplemented with 2 mM glutamine (pH = 7.4) following the sequential injection of glucose (10 mM), oligomycin (1 μM), and 2-DG (50 mM). Calculations were as follows: Glycolysis = Maximum rate measurement before oligomycin injection–Last rate measurement before glucose injection, representing basal glycolysis level; Glycolytic Capacity = Maximum rate measurement after oligomycin injection–Last rate measurement before glucose injection, representing maximum glycolysis level.

### CircRNA microarray analysis

Total RNAs extracted from ZCRB1-transfected U251 and U373 cells were quantified using the NanoDrop2000 spectrophotometer. The sample preparation and microarray hybridization were performed according to the standard protocols of Arraystar. In brief, circRNAs were enriched from total RNAs by digesting and eliminating linear RNAs with RNase R. Then, the enriched circRNAs were amplified and transcribed into fluorescent cRNAs by utilizing the Arraystar Super RNA Labeling Kit (Arraystar, Rockville, MD). The labeled cRNAs were hybridized onto the human circular RNA array V2.0 (Arraystar, Rockville, MD) and incubated at 65 °C for 17 h. After washing slides, the arrays were scanned by Agilent microarray scanner.

### RNase R digestion and actinomycin D treatment

RNase R digestion and actinomycin D treatment were both performed to confirm the stability of circHEATR5B in GBM cells. For RNase R digestion, 2 mg total RNAs were incubated with 3 U/mg RNase R (Lucigen, Madison, WI) at 37 °C for 30 min. For actinomycin D treatment, cells were treated with 2 mg/ml actinomycin D (NobleRyder, Beijing, China) and harvested after incubation for 0, 4, 8, 12, 24 h. After treatment with RNase R or actinomycin D, qRT–PCR assays were conducted to determine the expressions of circHEATR5B and HEATR5B mRNA.

### Nascent RNA capture assay

Nascent RNAs were prepared using the Click-iT Nascent RNA Capture Kit (Thermo Fisher, Waltham, MA) following the manufacture’s instruction. In brief, ZCRB1-transfected cells were incubated with 0.5 mM 5-ethynyl uridine for 15 min, and then EU-labeled RNAs were isolated using TRIzol reagent and biotinylated by Click reaction. The biotinylated RNAs were captured by streptavidin magnetic beads and reverse-transcribed into cDNA for the qRT–PCR analysis.

### RNA Immunoprecipitation assay (RIP)

EZ-Magna RIP™ RNA-Binding Protein Immunoprecipitation Kit (Millipore, Billerica, MA) was utilized for RIP assay. 100 μl lysates of ZCRB1-upregulated U251 cells (2 × 10^7^) were incubated with 50 μl magnetic beads coupled with 5 μg anti-ZCRB1 overnight at 4 °C. Mouse IgG antibody (Santa Cruz Cat# sc-2025) was used as the negative control. The immunoprecipitated RNA was isolated by proteinase K and analyzed by qRT–PCR assays. The primers are given in Additional file 2: Table S2.

### RNA pull-down assay

Pierce™ Magnetic RNA-Protein Pull-Down Kit (Thermo Fisher, Waltham, MA) was used for RNA pull-down assays. The flanking sequences and back-splicing junction of circHEATR5B were synthesized segmentally and biotinylated by Pierce RNA 3′ Desthiobiotinylation Kit. Bio1 and Bio3, the probes synthesized by 1 kb flanking sequences upstream and downstream from circHEATR5B exons, respectively; Bio2 and Bio4, the probes synthesized by 1–2 kb flanking sequences upstream and downstream from circHEATR5B exons, respectively; Bio5, 1 kb sequences across circHEATR5B junction. Then, biotinylated probes captured by streptavidin magnetic beads were incubated with the lysates of ZCRB1-upregulated U251 cells for 1 h at 4 °C. Finally, the pull-down proteins were detected by western blot assays after elution for 30 min at 37 °C.

### Fluorescence in situ hybridization assay (FISH)

FISH assays were performed using the RNA FISH assay kit (GenePharma, Shanghai, China). U251 and U373 cells were fixed on slides in 100 μl 4% paraformaldehyde at room temperature for 15 min. Then, the slides were treated with 100 μl 0.1% Buffer A (TritonX-100) for 15 min at room temperature. After washed twice with PBS, the slides were treated with 100 μl 2 × Buffer C (Saline-Sodium Citrate buffer) for 30 min at 37 °C and then incubated with 100 μl circHEATR5B probe (5′-Cy3-CGAUUUCAACCUGGUUGAGAAUAUUCCAGG-3′, red-labeled, GenePharma, Shanghai, China) overnight at 37 °C away from the light. The next day, after sequentially washed by 100 μl 0.1% Buffer F (Tween 20), 100 μl 2 × Buffer C, and 100 μl 1 × Buffer C, the slides were stained with 100 μl DAPI for 20 min away from the light. Finally, fluorescence images were captured under laser confocal microscopy.

### Nuclear and cytoplasmic extraction

Nuclear and cytoplasmic fractions were isolated using the PARIS™ kit (Thermo Fisher, Waltham, MA). Briefly, U251 and U373 cells were lysed by Cell Fractionation Buffer for 10 min on ice. Then, the supernatants were collected as the cytoplasmic fractions after centrifugation at 4 °C and 500×g for 3 min. Finally, the pellets were lysed by Cell Disruption Buffer to collect the nuclear fractions.

### Dual-luciferase reporter assay

The reporter vector constructions were carried out by inserting full-length, truncated, or mutated IRES into dual-luciferase reporter vectors. 293 T cells were transfected with constructed reporter vectors and the relative luciferase activities were detected 48 h after transfection using the Dual-Luciferase® Reporter Assay System Kit (Promega, Madison, WI) following the manufacturer’s protocol.

### Pyruvate kinase enzymatic activity assay

Pyruvate kinase enzymatic activity was measured using the pyruvate kinase assay kit (Nanjing Jiancheng, Jiangsu, China). Briefly, cell lysates were prepared and the protein concentration was measured. For the colorimetric assay, absorbance was measured at 340 nm at the 30 s after adding reagents to read A_1_ and measured again at the 15 min 30 s after incubating at 37 °C for 15 min to read A_2_. Relative pyruvate kinase enzymatic activity was calculated by the ratio of (A_1_–A_2_)/(protein concentration).

### Co-immunoprecipitation assay (Co-IP)

Co-IP assays were performed using the Pierce Co-immunoprecipitation (Co-IP) Kit (Thermo Fisher, Waltham, MA) following the manufacturer’s instructions. Cell lysates were prepared and incubated with AminoLink Plus Coupling Resin immobilized primary antibody overnight at 4 °C. Then, the samples were washed three times with 200 μl Wash Buffer and eluted with Elution Buffer for 5 min. The eluates were finally analyzed by western blot assays.

### GST pull-down assay

Prokaryotic expression plasmids fused with FLAG or GST tag were constructed including FLAG-HEATR5B-881aa, GST-JMJD5(-WT), GST-JMJD5-S361A. These plasmids were transformed into *E. coli* competent cell BL21 (TaKaRa, Liaoning, China) and protein expression was induced by 0.8 mM IPTG (Solarbio, Beijing, China) at 25 °C and 200 rpm for 6 h. Then, cells were lysed, sonicated, and centrifuged. The proteins were purified using the BeyoMag™ anti-Flag Magnetic Beads and BeyoGold™ GST-tag Purification Resin (Beyotime, Shanghai, China) according to the manufacturer’s procedure. For GST pull-down assay, purified FLAG-HEATR5B-881aa was incubated with purification resin coupled with GST-JMJD5 protein at 4 °C overnight. Then protein complexes were eluted by Elution Buffer and then subjected to SDS-PAGE and analyzed by western blot assays.

### In vitro kinase assay

In brief, 10 μg purified GST, GST-JMJD5(-WT), or GST-JMJD5-S361A proteins were incubated with 5 μg recombinant active HEATR5B-881aa in 50 μl kinase buffer (Cell Signaling Technology, Danvers, MA) containing 5 μCi [γ-^32^P] ATP (PerkinElmer, Waltham, MA) for 30–60 min at 30 °C. The kinase reaction was terminated by adding 12.5 μl 5 × SDS-PAGE loading buffer and boiling for 10 min. Then, the samples were resolved by SDS-PAGE gel electrophoresis. Radioactive signals were detected by autoradiography through a phosphor screen. The gels were subjected to Coomassie brilliant blue staining and destaining to visualize the protein bands.

### Mass spectrometry analysis

SDS-PAGE gel electrophoresis separated proteins and the targeted protein bands were excised from the gel. After elution, reduction and alkylation, the proteins were digested at 37 °C overnight. Peptides were collected, desalted and analyzed by timsTOF pro mass spectrometer (Bruker, Bremen, Germany). Sequence and site identification were analyzed using NCBI nonredundant protein database with Mascot Daemon.

### Cycloheximide chase assay

Cycloheximide, a protein synthesis inhibitor, was used to determine JMJD5 half-life. U251 cells transfected with JMJD5-WT, JMJD5-S361A, or JMJD5-S361E were treated with 100 mg/ml cycloheximide (NobleRyder, Beijing, China) and collected in 0, 2, 4, 8, 10 h. Then, total proteins were prepared and detected by western blot assays.

### Tumor xenografts in nude mice

Four-week-old nude mice (BALB/c) were purchased from Beijing Vital River Laboratory for in vivo study. The experiments were conducted strictly following the protocols approved by the Ethics Committee of China Medical University. The stably transfected U251 and U373 cells were selected and divided into five groups. For subcutaneous xenografts, 3 × 10^5^ cells were subcutaneously injected into the right flank of each nude mouse. Tumor volumes were measured every 4 days and calculated by formula: volume (mm^3^) = length×width^2^/2. The mice were sacrificed and then xenograft tumors were separated at the endpoints. For orthotopic xenografts, nude mice were injected with 3 × 10^5^ cells stereoscopically into the right striatum. The surviving numbers of nude mice were recorded, and Kaplan-Meier curves were used for survival analysis.

### Statistical analysis

All experimental data were indicated as mean ± SD. GraphPad Prism 5.01 was used for statistical analysis. Comparison between groups was analyzed by Student’s t-test, one-way ANOVA, or two-way ANOVA. Statistical significance was determined by *P*-value< 0.05.

## Results

### ZCRB1 overexpression inhibited glycolysis and proliferation in GBM cells

In our study, differentially expressed genes between GBM and NBTs were screened by RNA-seq (Additional file 1: Fig. S1A). Then, to screen glycolysis-inhibiting RBPs, we transiently silenced the top ten downregulated RBPs listed in Additional file 2: Table S3 in GBM cells. Given that aerobic glycolysis is characterized by the intense conversion of glucose to lactate, we detected the effects on glucose consumption and lactate production, and selected ZCRB1 for further study (Additional file 1: Fig. S1B).

**Fig. 1:**
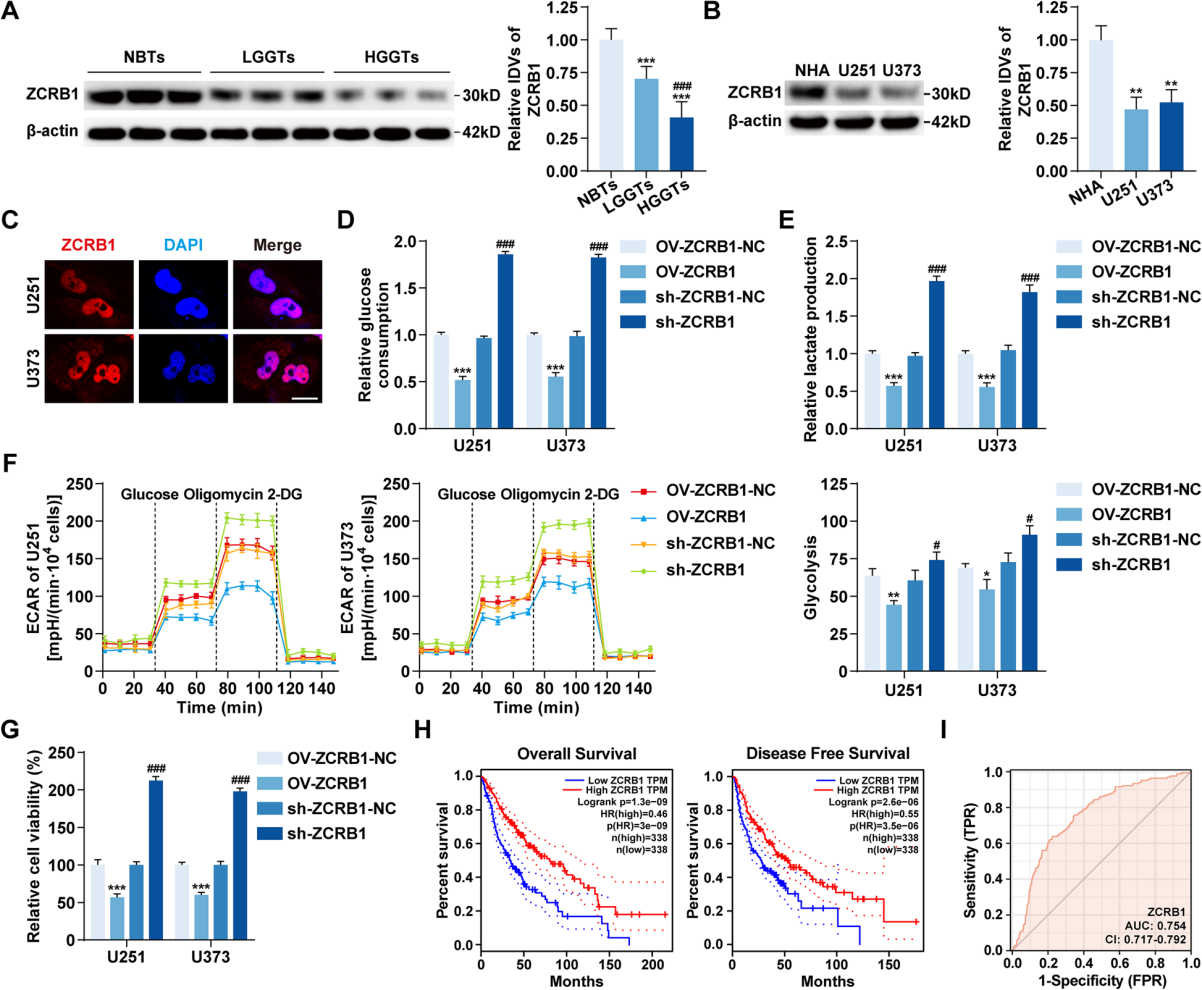
The expression of ZCRB1 and its effects on glycolysis and proliferation in GBM. **A** Western blot assays were used to detect the expression of ZCRB1 protein in non-tumor brain tissues (NBTs), low-grade glioma tissues (LGGTs), and high-grade glioma tissues (HGGTs). Data are presented as the mean ± SD (*n* = 9, each group). ^***^P < 0.001 vs. NBTs group; ^###^*P* < 0.001 vs. LGGTs group by one-way ANOVA. IDVs, integrated density values. **B** The expression of ZCRB1 protein in normal human astrocyte (NHA) and GBM cell lines (U251 and U373). Data are presented as the mean ± SD (*n* = 3, each group). ^**^P < 0.01 vs. NHA group by one-way ANOVA. **C** IF assays were used to detect the localization of ZCRB1 protein in U251 and U373 cells. Red, ZCRB1; blue, DAPI nuclear staining. Scale bars, 10 μm. **D **and** E** The effects of ZCRB1 on glucose consumption (**D**) and lactate production (**E**) in U251 and U373 cells. **F** Extracellular acidification rate (ECAR) was measured to demonstrate the effects of ZCRB1 on glycolysis in U251 and U373 cells and the glycolysis was calculated. **G** Cell viability assays were used to detect the effects of ZCRB1 on proliferation in U251 and U373 cells. Data are presented as the mean ± SD (*n* = 3, each group). ^*^P < 0.05, ^**^P < 0.01, ^***^P < 0.001 vs. OV-ZCRB1-NC group; ^#^*P* < 0.05, ^###^*P* < 0.001 vs. sh-ZCRB1-NC group by one-way ANOVA. **H** Kaplan–Meier analysis of the relationship of ZCRB1 expression with overall survival and disease-free survival of glioma patients through GEPIA database. **I** ROC curve of ZCRB1 mRNA expression in glioma cohort of TCGA database

Compared with NBTs, ZCRB1 mRNA and protein were both significantly downregulated in GBM tissues, and their expressions decreased even further as the pathological grades increased (Additional file 1: Fig. S1C, Fig. 1A). Among the four GBM cell lines, ZCRB1 mRNA was most significantly downregulated in U251 and U373 cells (Additional file 1: Fig. S1D), which were selected for subsequent studies. In U251 and U373 cells, ZCRB1 protein was also significantly downregulated (Fig. 1B). In addition, IF assays showed strong nuclear localization of ZCRB1 (Fig. 1C).

To assess the effects of ZCRB1 on glycolysis and proliferation in GBM cells, we constructed ZCRB1 stable overexpression or knockdown cells with the transfection efficiency confirmed by qRT–PCR and western blot assays (Additional file 1: Fig. S1E and F). The results showed that ZCRB1 overexpression significantly reduced glucose consumption and lactate production in U251 and U373 cells (Fig. 1D and E). Moreover, we performed Seahorse XF glycolytic stress assays to detect the ECAR and found that ZCRB1 overexpression significantly inhibited the basal glycolysis and glycolytic capacity in U251 and U373 cells (Fig. 1F, Additional file 1: Fig. S1G). The results of cell viability assays showed that the proliferation of U251 and U373 cells was markedly reduced by ZCRB1 overexpression (Fig. 1G). Conversely, ZCRB1 knockdown promoted glycolysis and proliferation in U251 and U373 cells (Fig. 1D-G). In the GEPIA database [31], low expression of ZCRB1 was correlated with worse overall survival and disease-free survival of glioma patients (Fig. 1H). To evaluate the diagnostic value of ZCRB1, receiver operating characteristic (ROC) curve analysis was performed, and the associated area under the curve was 0.754 (Fig. 1I).

### CircHEATR5B existed with low expression and inhibited glycolysis and proliferation in GBM cells

CircRNA microarray analysis was conducted in ZCRB1-upregulated GBM cells, and among the top ten upregulated circRNAs listed in Additional file 2: Table S4, circ_0054048 was found to be the most significantly upregulated, as validated by qRT–PCR assays (Additional file 1: Fig. S2A). Correlation analysis of the qRT–PCR assay results revealed a significantly positive correlation between ZCRB1 and circ_0054048 expression (Additional file 1: Fig. S2B).

**Fig. 2:**
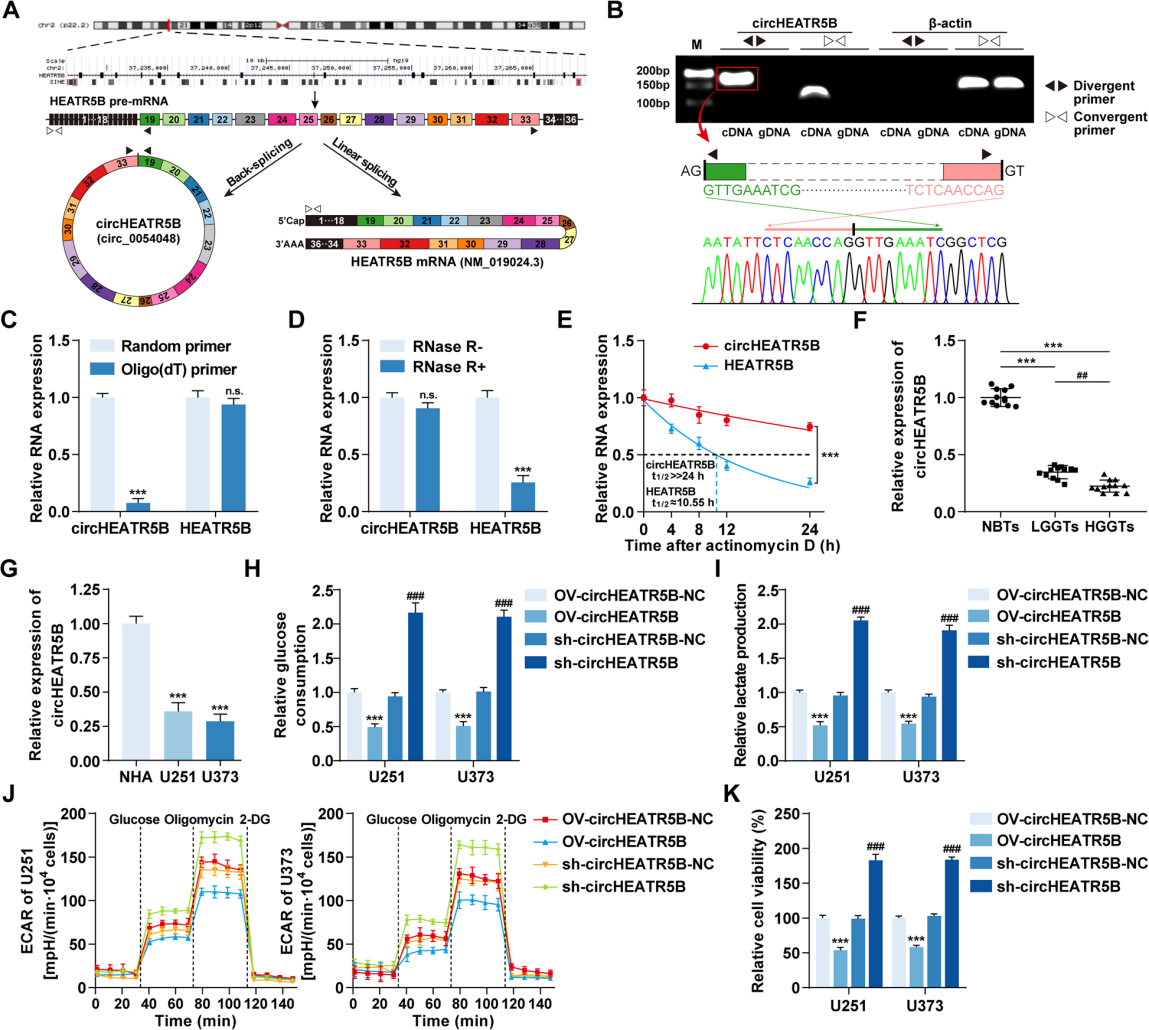
Identification of circHEATR5B and its expression and effects on glycolysis and proliferation in GBM. **A** Schematic illustration of the annotated genomic region of *HEATR5B* gene and the putative different RNA splicing forms. **B** Upper panel, the presence of circHEATR5B in cDNA and gDNA samples from U251 cells was detected using divergent and convergent primers, β-actin served as a linear RNA control. Lower panel, Sanger sequencing validated junction sequences. **C** The circHEATR5B and HEATR5B mRNA levels in U251 cDNAs reverse-transcribed by random or oligo (dT) primers. Data are presented as the mean ± SD (*n* = 3, each group). n.s. (not significant), ^***^P < 0.001 vs. random primer group by Student’s t-test. **D** The circHEATR5B and HEATR5B mRNA levels in U251 total RNAs after RNase R treatment. Data are presented as the mean ± SD (*n* = 3, each group). n.s., ^***^P < 0.001 vs. RNase R- group by Student’s t-test. **E** Half-lives of circHEATR5B and HEATR5B mRNA in U251 cells were analyzed after actinomycin D treatment. Data are presented as the mean ± SD (*n* = 3, each point). ^***^P < 0.001 vs. HEATR5B group by two-way ANOVA. **F** The expression of circHEATR5B in NBTs, LGGTs, and HGGTs groups. Data are presented as the mean ± SD (*n* = 12, each group). ^***^P < 0.001 vs. NBTs group; ^##^*P* < 0.01 vs. LGGTs group by one-way ANOVA. **G** The expression of circHEATR5B in NHA, U251, and U373 cells. Data are presented as the mean ± SD (*n* = 3, each group). ^***^P < 0.001 vs. NHA group by one-way ANOVA. **H and I** The effects of circHEATR5B on glucose consumption (H) and lactate production (I) in U251 and U373 cells. **J** The effects of circHEATR5B on ECAR in U251 and U373 cells. **K** The effects of circHEATR5B on proliferation in U251 and U373 cells. Data are presented as the mean ± SD (*n* = 3, each group). ^***^P < 0.001 vs. OV-circHEATR5B-NC group; ^###^*P* < 0.001 vs. sh-circHEATR5B-NC group by one-way ANOVA

Based on the circBase database [32], circHEATR5B (circ_0054048) was theoretically back-spliced by exons 19–33 of the *HEATR5B* gene (Fig. 2A). Then, we designed the divergent and convergent primers listed in Additional file 2: Table S5 to detect the endogenous formation of circHEATR5B in GBM cells. The results indicated that only divergent primers could amplify circHEATR5B from cDNA but not gDNA, which excluded the possibility of genomic rearrangement and trans-splicing or PCR artifacts, and the back-splicing junction sequences were further confirmed by Sanger sequencing (Fig. 2B). In addition, qRT–PCR assays were performed using cDNA reverse-transcribed by either random or oligo (dT) primers, and circHEATR5B was almost undetectable in the oligo (dT) primer group (Fig. 2C). Moreover, circHEATR5B levels in total RNAs showed no significant differences after RNase R treatment, and circHEATR5B had a longer half-life than HEATR5B mRNA (Fig. 2D and E).

CircHEATR5B was significantly reduced in GBM tissues and cells and had a negative correlation with the pathological grades of glioma (Fig. 2F and G), but HEATR5B mRNA levels showed no significant differences (Additional file 1: Fig. S2C and D). In addition, ZCRB1 overexpression did not change HEATR5B mRNA expression (Additional file 1: Fig. S2E). Then, as shown in Additional file 1: Fig. S2F, we generated circHEATR5B stable overexpression or knockdown cells with the transfection efficiency confirmed by qRT–PCR assays (Additional file 1: Fig. S2F and G). A significant reduction in glucose consumption, lactate production, ECAR, and proliferation of U251 and U373 cells was induced by circHEATR5B overexpression, while circHEATR5B knockdown promoted glycolysis and proliferation in U251 and U373 cells (Fig. 2H-K, Additional file 1: Fig. S2H).

### ZCRB1 inhibited glycolysis and proliferation in GBM cells by promoting circHEATR5B formation

**Fig. 3:**
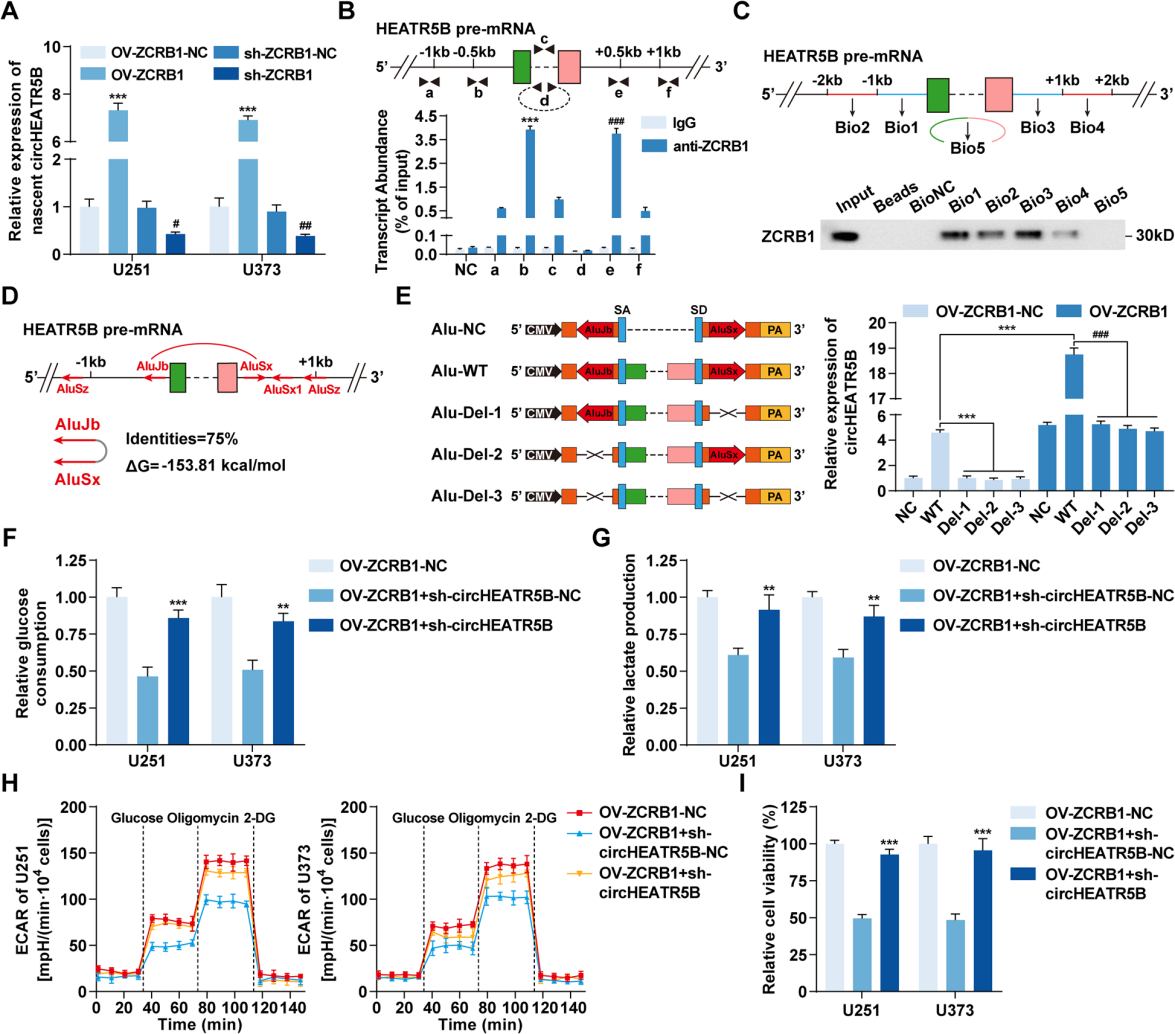
ZCRB1 promoted circHEATR5B formation, thereby inhibiting glycolysis and proliferation in GBM cells. **A** The expression of nascent circHEATR5B in response to ZCRB1 transfection in U251 and U373 cells. Data are presented as the mean ± SD (*n* = 3, each group). ^***^P < 0.001 vs. OV-ZCRB1-NC group; ^#^*P* < 0.05, ^##^*P* < 0.01 vs. sh-ZCRB1-NC group by one-way ANOVA. **B** Upper panel, primer (a-f) designs for RIP assays. Lower panel, the enrichment of ZCRB1 on circHEATR5B flanking regions was detected by RIP assays. β-actin served as the negative control. Data are presented as the mean ± SD (*n* = 3, each group). ^***^P < 0.001 vs. other anti-ZCRB1 groups except e; ^###^*P* < 0.001 vs. other anti-ZCRB1 groups except b by one-way ANOVA. **C** Upper panel, biotinylated probe (Bio1–5) designs for RNA pull-down assay. Lower panel, western blot assays showed the level of ZCRB1 pulled down by biotinylated probes. **D** Schematic of Alu elements in circHEATR5B flanking sequences. Red arrows indicated the polarity of Alu elements. Red arc lines indicated the only pair of inverted repeated Alu elements within 1 kb with their identities and minimum free energies (ΔG) listed below. **E** Left panel, schematic of plasmids with wild-type Alu elements (Alu-WT) and deletion constructs (Alu-Del-1/2/3). SA, splicing acceptor; SD, splicing donor. Right panel, the expression of circHEATR5B in U251 cells transfected with Alu plasmids and OV-ZCRB1 plasmids. Data are presented as the mean ± SD (*n* = 3, each group). ^***^P < 0.001 vs. Alu-WT in OV-ZCRB1-NC group; ^###^*P* < 0.001 vs. Alu-WT in OV-ZCRB1 group by one-way ANOVA. **F and G** The effects of ZCRB1 and circHEATR5B on glucose consumption (**F**) and lactate production (**G**) in U251 and U373 cells. **H** The effects of ZCRB1 and circHEATR5B on ECAR in U251 and U373 cells. **I** The effects of ZCRB1 and circHEATR5B on proliferation in U251 and U373 cells. Data are presented as the mean ± SD (*n* = 3, each group). ^**^P < 0.01, ^***^P < 0.001 vs. OV-ZCRB1 + sh-circHEATR5B-NC group by one-way ANOVA

To further reveal the mechanisms by which ZCRB1 increased circHEATR5B expression, we analyzed the expression of nascent circHEATR5B and the half-life of circHEATR5B in ZCRB1-upregulated or ZCRB1-downregulated cells. The results showed that the half-life of circHEATR5B was not significantly changed by ZCRB1 transfection (Additional file 1: Fig. S3A). However, nascent circHEATR5B significantly increased when ZCRB1 was upregulated and decreased when ZCRB1 was downregulated (Fig. 3A). ZCRB1 was predicted to bind to circHEATR5B flanking sequences by the RBPmap database [33] (Additional file 1: Fig. S3B). As shown in Fig. 3B (upper panel), primers were designed to quantify the enrichment of ZCRB1 on circHEATR5B flanking regions by RIP assays. We found that ZCRB1 mainly bound to exon-adjacent regions rather than farther regions or circHEATR5B itself (Fig. 3B, lower panel). Moreover, the flanking segments and back-splicing junction were synthesized and labeled with biotin for RNA pull-down assays, as shown in Fig. 3C (upper panel). The results showed that Bio1/3 probes pulled down more ZCRB1 protein than Bio2/4 probes, and the Bio5 probe did not pull down ZCRB1 protein (Fig. 3C, lower panel).

The UCSC database [34] search revealed that a pair of highly reverse complementary Alu elements, AluJb and AluSx (75% identity, Fig. 3D), existed in the flanking sequences within 1 kb from circHEATR5B exons. To test whether ZCRB1 promoted circHEATR5B formation by facilitating the binding of AluJb and AluSx, different circHEATR5B expression plasmids were constructed by inserting the flanking sequences with wild-type Alu elements or a series of deletions into both sides of the circHEATR5B gene. After transfection into ZCRB1-upregulated U251 cells, qRT–PCR assay results showed that the plasmid with wild-type Alu elements, but not the deletion constructs, could generate circHEATR5B, which was greatly facilitated by ZCRB1 overexpression (Fig. 3E). Furthermore, ZCRB1-upregulated cells were transfected with sh-circHEATR5B plasmids. CircHEATR5B knockdown rescued the ZCRB1 upregulation-induced inhibitory effects on glycolysis and proliferation in U251 and U373 cells (Fig. 3F-I, Additional file 1: Fig. S3C).

### CircHEATR5B encoded an 881 amino acid protein, HEATR5B-881aa, in GBM cells

**Fig. 4:**
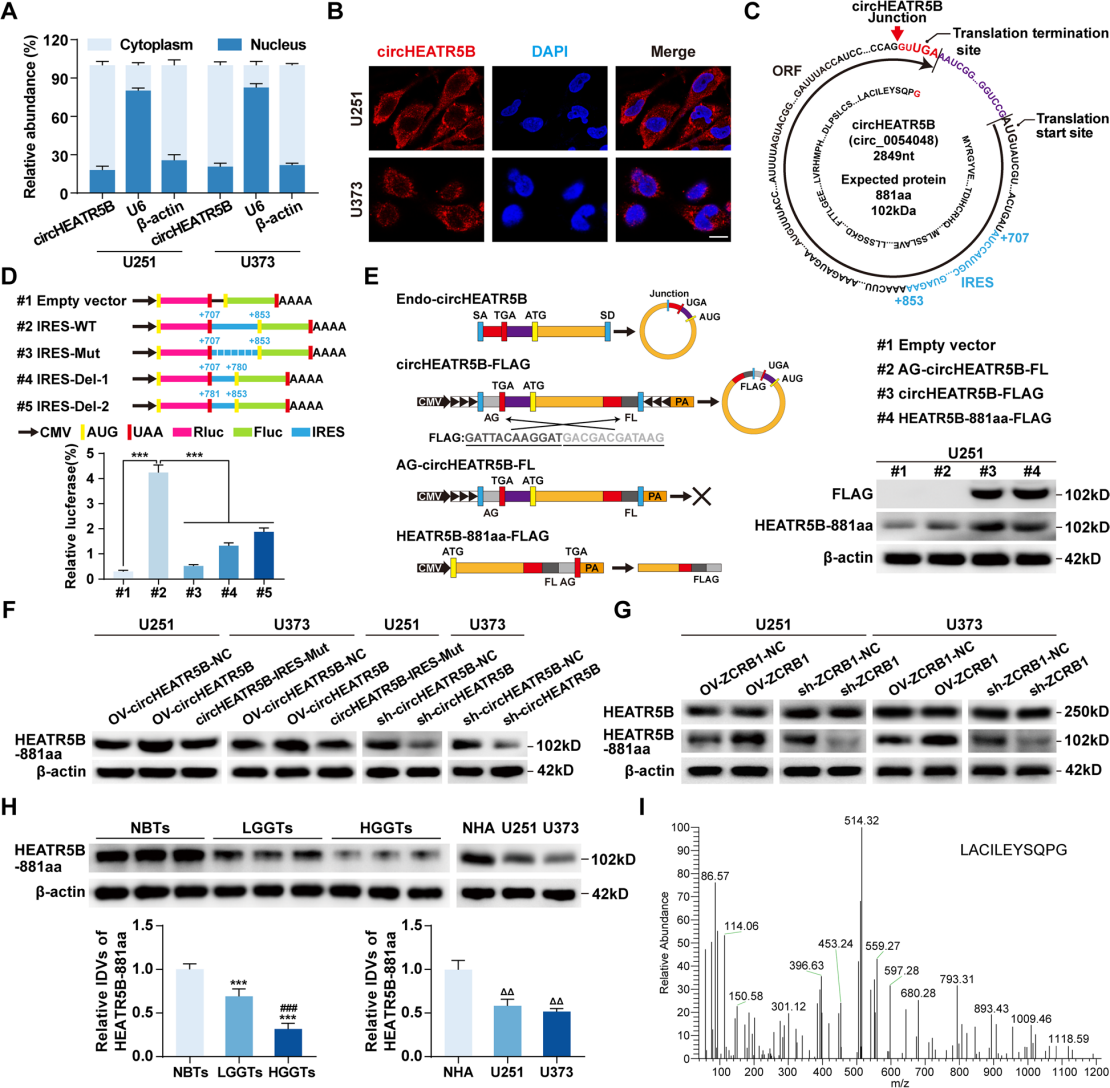
CircHEATR5B encoded a novel protein termed HEATR5B-881aa. **A** The cytoplasmic and nuclear distribution of circHEATR5B in U251 and U373 cells. β-actin and U6 served as cytoplasmic and nuclear RNA markers, respectively. **B** The localization of circHEATR5B in U251 and U373 cells by FISH assays. Red, circHEATR5B; blue, DAPI nuclear staining. Scale bars, 10 μm. **C** Schematic of putative protein encoded by circHEATR5B. Red arrow, the junction site; black arrow, the ORF sequences; red sequence, the unique amino acid encoded by the junction-spanning ORF; blue sequences, the putative IRES sequences. **D** Upper panel, schematic of plasmids with wild-type, mutated, or truncated IRES inserted between the Rluc stop codon and Fluc start codon. Lower panel, the luciferase activity of Fluc/Rluc was detected by dual-luciferase reporter assays. Data are presented as the mean ± SD (*n* = 3, each group). ^***^P < 0.001 vs. #2 group by one-way ANOVA. **E** Left panel, plasmid constructions for detection of the coding ability of circHEATR5B-ORF. Right panel, FLAG tag and HEATR5B-881aa antibodies were used to detect HEATR5B-881aa expression in U251 cells transfected with plasmids. **F** The expression of HEATR5B-881aa in response to circHEATR5B transfection in U251 and U373 cells. **G** The expressions of HEATR5B protein and HEATR5B-881aa in response to ZCRB1 transfection in U251 and U373 cells. **H** The expression of HEATR5B-881aa in NBTs, LGGTs, and HGGTs groups. Data are presented as the mean ± SD (*n* = 9, each group). ^***^P < 0.001 vs. NBTs group; ^###^*P* < 0.001 vs. LGGTs group by one-way ANOVA. The expression of HEATR5B-881aa in NHA, U251, and U373 cells. Data are presented as the mean ± SD (*n* = 3, each group). ^ΔΔ^*P* < 0.01 vs. NHA group by one-way ANOVA. **I** Mass spectrometry analysis of HEATR5B-881aa in GBM cells

To explore circHEATR5B functions, we first detected the localization of circHEATR5B. qRT–PCR assays after nucleoplasm separation showed that circHEATR5B levels in the cytoplasm were significantly higher than that in the nucleus (Fig. 4A). FISH assays showed that circHEATR5B was mainly located in the cytoplasm of U251 and U373 cells (Fig. 4B).

The circRNADb database search suggested that circHEATR5B contained one open reading frame (ORF) and two internal ribosome entry sites (IRESs) and might encode an 881 amino acid protein (Additional file 1: Fig. S4A, Fig. 4C). Two IRES elements were inserted into dual-luciferase reporter vectors to detect circHEATR5B-IRES activity, and IRES-1 was selected due to its significantly higher activity (Additional file 1: Fig. S4B). Then, we further mutated and truncated IRES-1, and the luciferase assay results showed that wild-type IRES induced the highest Fluc/Rluc activity (Fig. 4D). To detect the coding ability of circHEATR5B-ORF, as shown in Fig. 4E (left panel), we moved the junction of the circHEATR5B gene to the stop codon, and the FLAG tag gene was divided into two halves and inserted crosswise on both sides of the junction to construct the circHEATR5B-FLAG plasmid. Only if the new ORF formed by cyclization was translated could the FLAG tag be detected. In addition, we also removed the downstream flanking repeat sequences to construct the AG-circHEATR5B-FL plasmid as a negative control and inserted an intact FLAG tag before the stop codon of the HEATR5B-881aa sequences to construct the HEATR5B-881aa-FLAG plasmid as a positive control. Then, we transfected these plasmids into U251 cells and detected their potential translation products. Similar to the positive control, the putative protein encoded by circHEATR5B was detected at the same molecular mass as the FLAG tag protein by a custom antibody targeting HEATR5B-881aa (antigen design shown in Additional file 1: Fig. S4C) in the circHEATR5B-FLAG group (Fig. 4E, left panel).

In U251 and U373 cells, we found that HEATR5B-881aa decreased with circHEATR5B knockdown and increased with circHEATR5B overexpression (Fig. 4F). We then constructed a circHEATR5B plasmid with an IRES mutation, which had the same circHEATR5B overexpression efficiency as the OV-circHEATR5B plasmid (Additional file 1: Fig. S4D) but reversed the circHEATR5B-induced increase in HEATR5B-881aa (Fig. 4F, Additional file 1: Fig. S4E). We also detected HEATR5B-881aa in ZCRB1-upregulated or ZCRB1-downregulated U251 and U373 cells. ZCRB1 increased HEATR5B-881aa expression without changing HEATR5B protein expression (Fig. 4G, Additional file 1: Fig. S4F). In GBM tissues and cells, HEATR5B-881aa was significantly expressed at low levels and had a negative correlation with the pathological grades of glioma (Fig. 4H), but HEATR5B protein levels showed no significant differences (Additional file 1: Fig. S4G). Finally, protein bands were excised for mass spectrometry analysis at the molecular mass recognized by the HEATR5B-881aa antibody, and the unique sequence of HEATR5B-881aa (LACILEYSQPG) was verified (Fig. 4I).

### HEATR5B-881aa, but not circHEATR5B, exerted inhibitory effects on GBM cells

**Fig. 5:**
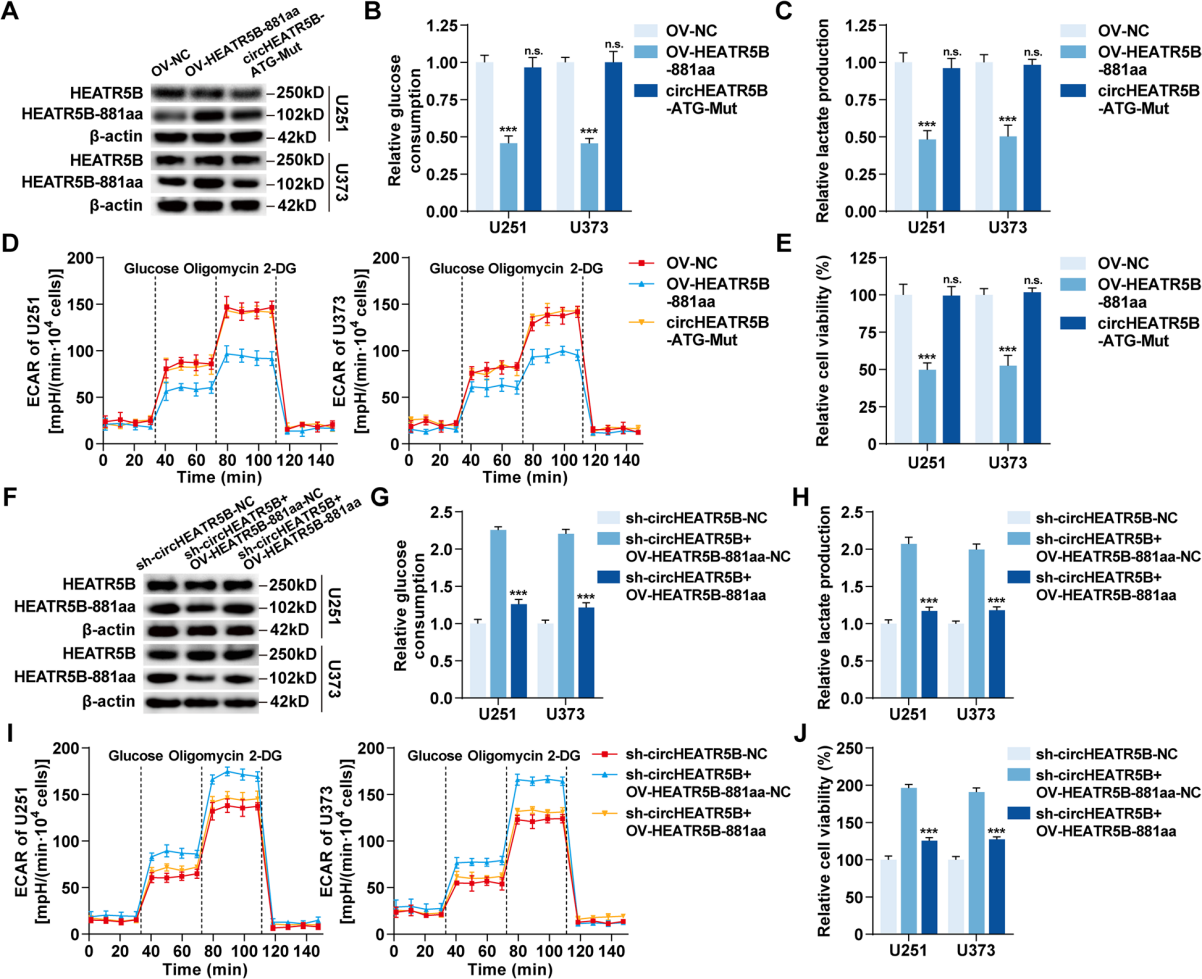
HEATR5B-881aa, but not circHEATR5B, exerted inhibitory effects on GBM cells. **A** HEATR5B protein and HEATR5B-881aa were detected by western blot assays in U251 and U373 cells transfected with OV-HEATR5B-881aa or circHEATR5B-ATG-Mut plasmid. **B** and **C** The effects of HEATR5B-881aa on glucose consumption (**B**) and lactate production (**C**) in U251 and U373 cells. **D** The effects of HEATR5B-881aa on ECAR in U251 and U373 cells. **E** The effects of HEATR5B-881aa on proliferation in U251 and U373 cells. Data are presented as the mean ± SD (*n* = 3, each group). n.s., ^***^P < 0.001 vs. OV-NC group by one-way ANOVA. **F** HEATR5B protein and HEATR5B-881aa were detected by western blot assays in U251 and U373 cells transfected with sh-circHEATR5B plus OV-HEATR5B-881aa plasmids. **G** and **H** The effects of circHEATR5B and HEATR5B-881aa on glucose consumption (**G**) and lactate production (**H**) in U251 and U373 cells. **I** The effects of circHEATR5B and HEATR5B-881aa on ECAR in U251 and U373 cells. **J** The effects of circHEATR5B and HEATR5B-881aa on proliferation in U251 and U373 cells. Data are presented as the mean ± SD (*n* = 3, each group). ^***^P < 0.001 vs. sh-circHEATR5B + OV-HEATR5B-881aa-NC group by one-way ANOVA

To investigate the biological functions of HEATR5B-881aa, we upregulated HEATR5B-881aa using OV-HEATR5B-881aa plasmids and downregulated HEATR5B-881aa using sh-circHEATR5B plasmids without affecting HEATR5B protein in U251 and U373 cells (Fig. 5A and F, Additional file 1: Fig. S5A and C). HEATR5B-881aa upregulation significantly inhibited the glucose consumption, lactate production, ECAR, and proliferation in U251 and U373 cells, while HEATR5B-881aa downregulation had the opposite effects (Fig. 5B-E and G-J, Additional file 1: Fig. S5B and D).

To further exclude the possibility that circHEATR5B, but not HEATR5B-881aa, induced the above effects, we mutated the ATG start codon of circHEATR5B (Fig. 5A, Additional file 1: Fig. S5A) and found that it could not inhibit the glycolysis and proliferation in U251 and U373 cells (Fig. 5B-E, Additional file 1: Fig. S5B). Then, we restored HEATR5B-881aa expression in circHEATR5B-downregulated U251 and U373 cells using OV-HEATR5B-881aa plasmids (Fig. 5F, Additional file 1: Fig. S5C). HEATR5B-881aa re-expression rescued circHEATR5B knockdown-induced promoting effects on glycolysis and proliferation in U251 and U373 cells (Fig. 5G-J, Additional file 1: Fig. S5D).

### HEATR5B-881aa reduced JMJD5 expression, thereby inhibiting glycolysis and proliferation in GBM cells

**Fig. 6:**
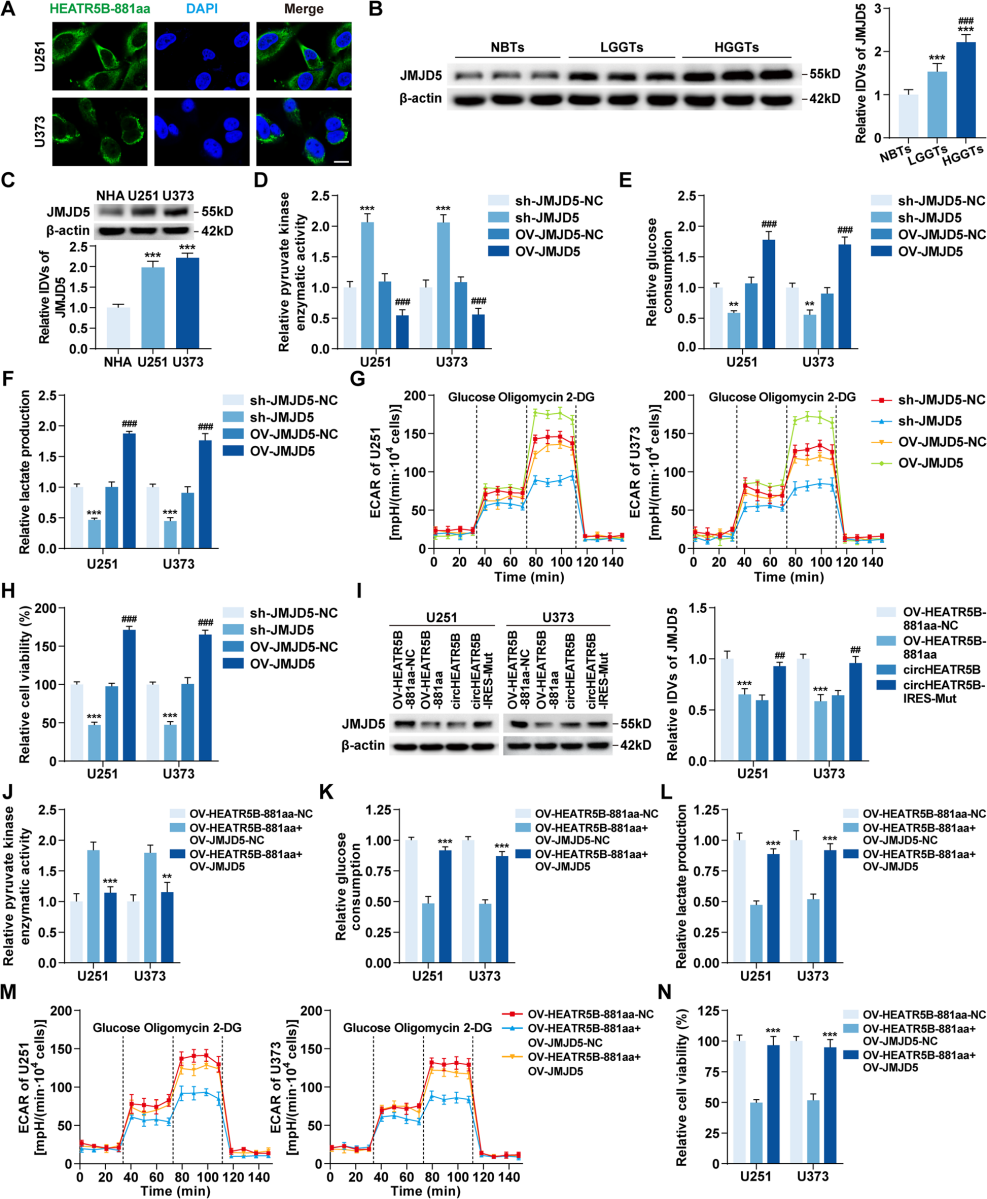
The expression and oncogenic role of JMJD5 in glycolysis and proliferation of GBM. **A** The localization of HEATR5B-881aa in U251 and U373 cells by IF assays. Green, HEATR5B-881aa; blue, DAPI nuclear staining. Scale bars, 10 μm. **B** The expression of JMJD5 protein in NBTs, LGGTs, and HGGTs groups. Data are presented as the mean ± SD (*n* = 9, each group). ^***^P < 0.001 vs. NBTs group; ^###^*P* < 0.001 vs. LGGTs group by one-way ANOVA. **C** The expression of JMJD5 protein in NHA, U251, and U373 cells. Data are presented as the mean ± SD (*n* = 3, each group). ^***^P < 0.001 vs. NHA group by one-way ANOVA. **D** The effects of JMJD5 on pyruvate kinase enzymatic activity in U251 and U373 cells. **E **and** F** The effects of JMJD5 on glucose consumption (**E**) and lactate production (**F**) in U251 and U373 cells. **G** The effects of JMJD5 on ECAR in U251 and U373 cells. **H** The effects of JMJD5 on proliferation in U251 and U373 cells. Data are presented as the mean ± SD (*n* = 3, each group). ^**^P < 0.01, ^***^P < 0.001 vs. sh-JMJD5-NC group; ^###^*P* < 0.001 vs. OV-JMJD5-NC group by one-way ANOVA. **I** The expression of JMJD5 protein in response to HEATR5B-881aa upregulation or downregulation in U251 and U373 cells. Data are presented as the mean ± SD (*n* = 3, each group). ^***^P < 0.001 vs. OV-HEATR5B-881aa-NC group; ^##^*P* < 0.01 vs. circHEATR5B group by one-way ANOVA. **J-L** The effects of HEATR5B-881aa and JMJD5 on pyruvate kinase enzymatic activity (**J**), glucose consumption (**K**), and lactate production (**L**) in U251 and U373 cells. **M** The effects of HEATR5B-881aa and JMJD5 on ECAR in U251 and U373 cells. **N** The effects of HEATR5B-881aa and JMJD5 on proliferation in U251 and U373 cells. Data are presented as the mean ± SD (*n* = 3, each group). ^**^P < 0.01, ^***^P < 0.001 vs. OV-HEATR5B-881aa + OV-JMJD5-NC group by one-way ANOVA

IF assays revealed that HEATR5B-881aa was mainly localized in the cytoplasm (Fig. 6A). To reveal the functions of HEATR5B-881aa in the cytoplasm, HEATR5B-881aa–interacting proteins were coimmunoprecipitated for mass spectrometry analysis in the cytoplasmic fractions of U251 cells expressing FLAG-HEATR5B-881aa. Among the candidates shown in Additional file 1: Fig. S6A, JMJD5 was selected for further study. The binding affinity of HEATR5B-881aa and JMJD5 was predicted with a small dissociation constant by the PPA-Pred2 server [35] (Additional file 1: Fig. S6B). In addition, in silico docking performed by the ZDOCK server [36] showed that HEATR5B-881aa docked with JMJD5 (Additional file 1: Fig. S6C).

In GBM tissues and cells, JMJD5 was significantly elevated and positively correlated with the pathological grades of glioma (Fig. 6B and C). We further constructed JMJD5 stable knockdown or overexpression cells with the transfection efficiency detected by qRT–PCR and western blot assays (Additional file 1: Fig. S6D and E). JMJD5 knockdown significantly increased pyruvate kinase enzymatic activity and reduced glycolysis and proliferation in U251 and U373 cells, whereas the converse was observed upon JMJD5 overexpression (Fig. 6D-H, Additional file 1: Fig. S6F).

Then, we found that JMJD5 protein decreased as HEATR5B-881aa increased and that it increased as HEATR5B-881aa decreased (Fig. 6I). However, JMJD5 mRNA levels did not change (Additional file 1: Fig. S6G). Moreover, we restored JMJD5 expression in HEATR5B-881aa-upregulated U251 and U373 cells using OV-JMJD5 plasmids and found that JMJD5 re-expression rescued the pyruvate kinase enzymatic activity enhancement and the glycolytic and proliferative inhibition mediated by HEATR5B-881aa overexpression (Fig. 6J-N, Additional file 1: Fig. S6H).

### HEATR5B-881aa phosphorylated JMJD5 on S361 and reduced JMJD5 stability

**Fig. 7:**
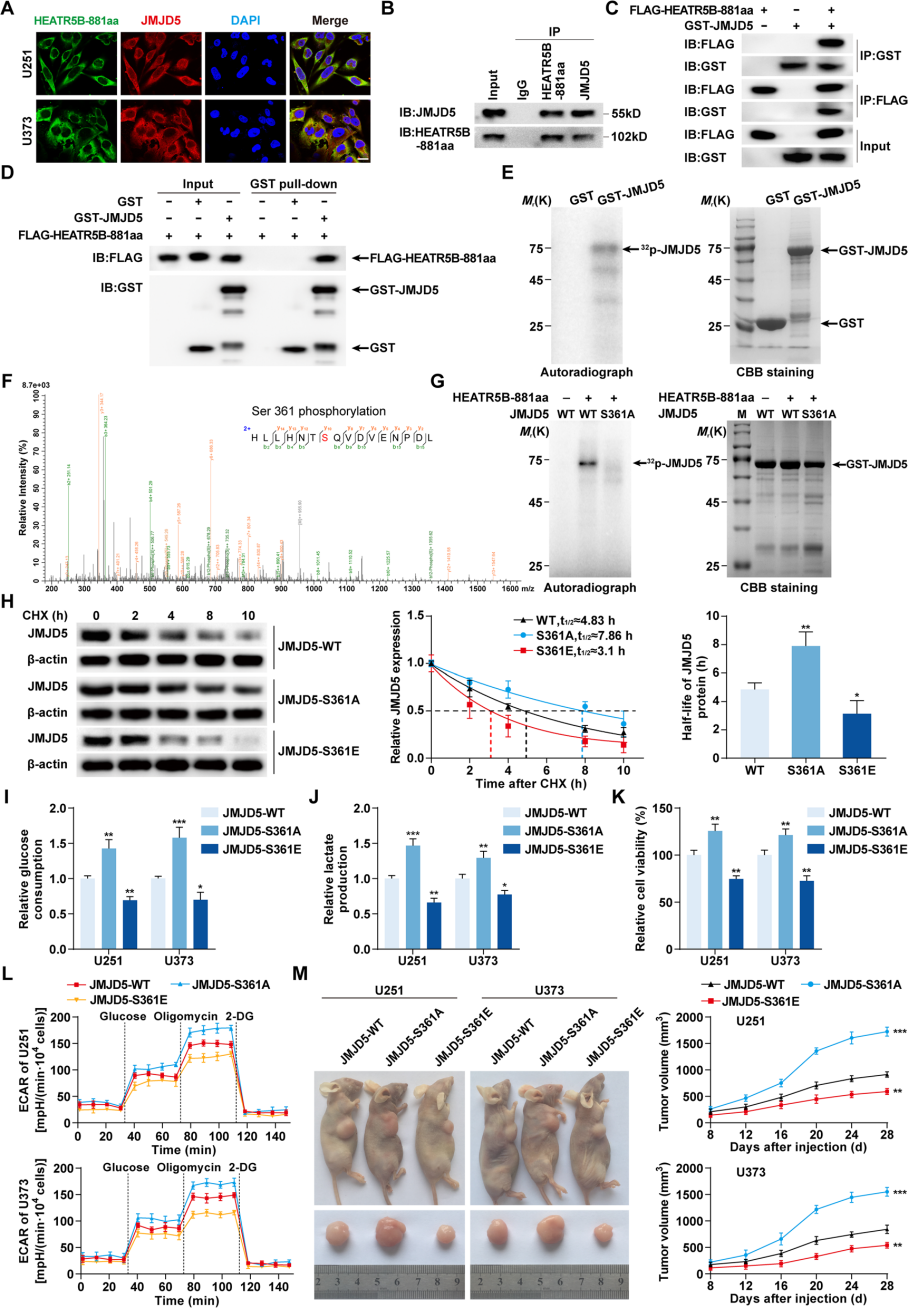
The effects of JMJD5 phosphorylated by HEATR5B-881aa on glycolysis and proliferation in GBM. **A** The colocalization of HEATR5B-881aa and JMJD5 in U251 and U373 cells by IF assays. Green, HEATR5B-881aa; red, JMJD5; blue, DAPI nuclear staining. Scale bars, 10 μm. **B** Lysates of U251 cells were subjected to immunoprecipitation (IP) and immunoblotting (IB) with HEATR5B-881aa and JMJD5 antibodies. **C** Lysates of 293 T cells transfected with FLAG-HEATR5B-881aa and GST-JMJD5 plasmids were subjected to IP and IB with FLAG tag and GST tag antibodies. **D** The direct interaction between HEATR5B-881aa and JMJD5 was confirmed by GST pull-down assays. GST protein functioned as a negative control. **E** Left panel, in vitro kinase assays were performed and detected by autoradiography (arrow, phosphorylated band). Right panel, proteins were visualized by Coomassie brilliant blue (CBB) staining. **F** The phosphorylated bands were subjected to mass spectrometry and S361 was identified. **G** In vitro kinase assays and CBB staining were conducted after S361 mutation. **H** The effects of S361 phosphorylation on JMJD5 stability were detected by Cycloheximide (CHX) chase assays. Data are presented as the mean ± SD (*n* = 3, each group). ^*^P < 0.05, ^**^P < 0.01 vs. JMJD5-WT group by one-way ANOVA. **I-K** The effects of JMJD5 phosphorylation on glucose consumption (**I**), lactate production (**J**), and proliferation (**K**) in U251 and U373 cells. Data are presented as the mean ± SD (*n* = 3, each group). ^*^P < 0.05, ^**^P < 0.01, ^***^P < 0.001 vs. JMJD5-WT group by one-way ANOVA. **L** The effects of JMJD5 phosphorylation on ECAR in U251 and U373 cells. **M** Subcutaneous xenograft nude mouse models using U251 and U373 cells transfected with JMJD5-WT, JMJD5-S361A, or JMJD5-S361E were established with tumor growth curves. Data are presented as the mean ± SD (*n* = 5, each group). ^**^P < 0.01, ^***^P < 0.001 vs. JMJD5-WT group by two-way ANOVA

To further investigate the mechanisms by which HEATR5B-881aa downregulated JMJD5 expression, IF assays were performed and showed that HEATR5B-881aa colocalized with JMJD5 in the cytoplasm of U251 and U373 cells (Fig. 7A). Co-IP assays indicated that endogenous HEATR5B-881aa bound to JMJD5 in U251 cells and FLAG-HEATR5B-881aa bound to GST-JMJD5 in 293 T cells (Fig. 7B and C). GST pull-down assays using purified FLAG-HEATR5B-881aa and GST-JMJD5 proteins proved that HEATR5B-881aa directly bound to JMJD5 in vitro (Fig. 7D).

In vitro kinase assays revealed that HEATR5B-881aa phosphorylated JMJD5 protein (Fig. 7E). By analyzing the phosphorylated band by mass spectrometry, the serine at position 361 (S361) of JMJD5 was identified as a HEATR5B-881aa–specific phosphorylation site (Fig. 7F), which was highly conserved in several species (Additional file 1: Fig. S7A). We mutated S361 to alanine (S361A), rendering it unable to be phosphorylated, and found that the phosphorylated band disappeared (Fig. 7G). Likewise, JMJD5-S361A had a lower phosphorylation level than wild-type JMJD5 as detected by a custom JMJD5-S361–specific phosphorylation antibody in vivo, while JMJD5-S361E, a mutant in which S361 was replaced with glutamate to imitate a phosphorylated status, had a higher phosphorylation level (Additional file 1: Fig. S7B). The S361 phosphorylation level increased as HEATR5B-881aa overexpression and JMJD5-S361A expression increased, but JMJD5-S361E expression decreased (Additional file 1: Fig. S7C and D). Cycloheximide chase assays found that the half-life of JMJD5-S361A significantly increased. However, the half-life of JMJD5-S361E significantly decreased (Fig. 7H), which was reversed by MG132 treatment (Additional file 1: Fig. S7E).

Subsequently, we transfected JMJD5-WT, JMJD5-S361A, and JMJD5-S361E plasmids into U251 and U373 cells and found that JMJD5 with low phosphorylation levels significantly increased glucose consumption, lactate production, proliferation, and ECAR in U251 and U373 cells and promoted xenograft tumor growth, while JMJD5 with high phosphorylation levels significantly inhibited these effects (Fig. 7I-M, Additional file 1: Fig. S7F).

### Overexpression of ZCRB1, circHEATR5B, and HEATR5B-881aa inhibited GBM growth in vivo and prolonged the survival time of nude mice

**Fig. 8:**
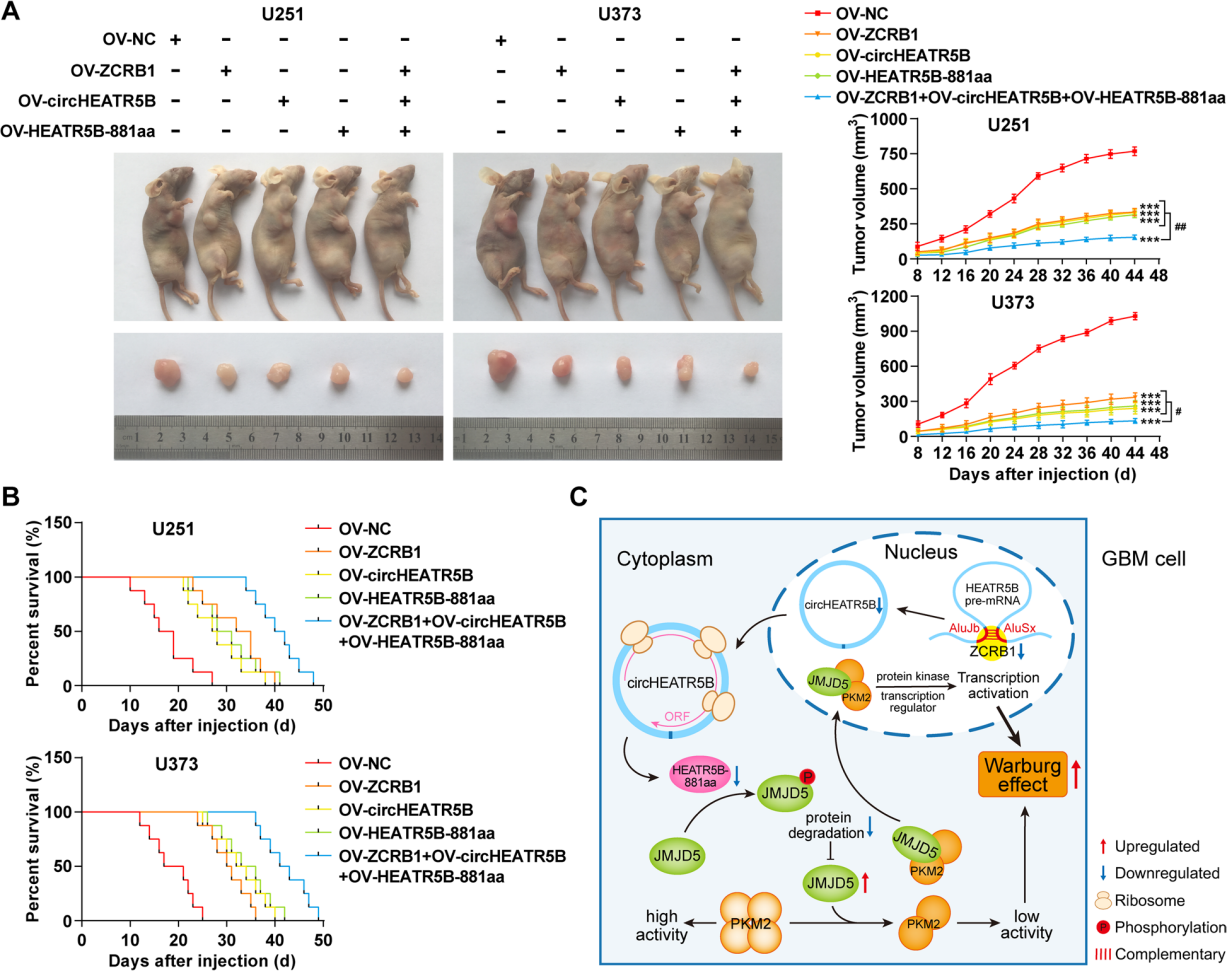
Tumor xenograft studies and mechanism diagram. **A** Left panel, the nude mice carrying subcutaneous xenografts in different groups and the dissected tumor samples from respective groups. Right panel, subcutaneous tumor growth curves. Data are presented as the mean ± SD (*n* = 5, each group). ^***^P < 0.001 vs. OV-NC group; ^#^*P* < 0.05, ^##^*P* < 0.01 vs. OV-ZCRB1+OV-circHEATR5B+OV-HEATR5B-881aa group by two-way ANOVA. **B** Survival curves of nude mice with orthotopic xenografts (*n* = 5, each group). **C** The schematic of the mechanisms of the ZCRB1/circHEATR5B/HEATR5B-881aa/JMJD5/PKM2 pathway in GBM cells

Finally, nude mouse xenograft models were used to clarify the in vivo tumor-suppressive effects of ZCRB1, circHEATR5B, and HEATR5B-881aa. The grouping is shown in Fig. 8A. Compared with the negative control group, subcutaneous xenograft tumor volumes were significantly reduced in the groups of ZCRB1, circHEATR5B, and HEATR5B-881aa overexpression, and the three-combined group resulted in the minimum tumor volume (Fig. 8A).

Survival analysis of nude mice with orthotopic xenograft tumors revealed that the groups of ZCRB1, circHEATR5B, and HEATR5B-881aa overexpression had longer survival time than the negative control group, and the three-combined group had the longest survival time (Fig. 8B). The mechanism is schematically presented in Fig. 8C.

## Discussion

RBPs have been reported to play an important regulatory role in tumor glycolysis [37]. Therefore, to screen for RBPs with regulatory effects on GBM glycolysis, we identified the top ten downregulated RBPs in GBM tissues by RNA-seq. Then, ZCRB1, which had the greatest inhibitory effects on glycolysis in GBM cells, was selected for further study. We confirmed that ZCRB1 was expressed at low levels in GBM tissues and cells and could inhibit glycolysis and proliferation in GBM cells. These results support that ZCRB1 functions as a negative regulator of GBM glycolysis and progression. Moreover, Kaplan–Meier survival curves and ROC curves indicate that ZCRB1 may be a useful prognostic and diagnostic biomarker for glioma patients.

Previous studies have indicated that circRNAs exert essential functions in GBM development [38–40]. To investigate whether ZCRB1 functioned by targeting circRNAs, we performed circRNA microarray analysis in ZCRB1-upregulated GBM cells and selected circHEATR5B (circ_0054048) as a potential target, whose existence was proven by PCR using divergent primers and Sanger sequencing. In addition, the inability of oligo (dT) primers to reverse-transcribe circHEATR5B indicates that circHEATR5B has no poly(A) tail. Moreover, the resistance to RNase R digestion and the long half-life of circHEATR5B indicate that it has a stable structure. Based on the above characteristics, we conclude that circHEATR5B exists in GBM cells in a circular form. In GBM, circHEATR5B was expressed at low levels and could suppress glycolysis, whereas HEATR5B mRNA levels showed no significant differences and could not be regulated by ZCRB1, indicating that it is circHEATR5B but not HEATR5B mRNA that exerts glycolytic inhibition in GBM cells under the regulation of ZCRB1. Similarly, circRPN2 inhibits aerobic glycolysis in hepatocellular carcinoma and represents a therapeutic target in hepatocellular carcinoma [41].

We found that ZCRB1 overexpression increased nascent circHEATR5B expression without changing its half-life, indicating that ZCRB1 upregulates circHEATR5B by promoting its formation rather than inhibiting its degradation. To elucidate the mechanisms by which ZCRB1 promoted circHEATR5B formation, we first predicted the binding of ZCRB1 and circHEATR5B flanking sequences by RBPmap database and further confirmed it experimentally. It has been reported that RBPs such as QKI and Mbl can promote circRNA formation by binding flanking sequences [42, 43]. Back-splicing of circularized exons usually requires regulatory elements with reverse complementary pairing in flanking sequences [44]. The UCSC database search further identified AluJb and AluSx in the ZCRB1-binding regions. Alu elements are short scattered repeat sequences that are essential for exon circularization [45, 46]. In this study, AluJb and AluSx were found to be indispensable for circHEATR5B formation, whose complementary pairing could be promoted by ZCRB1, thereby remarkably increasing the formation of splice variant circ_0054048 between AluJb and AluSx. In addition, circHEATR5B knockdown rescued ZCRB1-induced inhibitory effects. In conclusion, ZCRB1 plays an inhibitory role by facilitating circHEATR5B formation.

Previous studies have proven that circRNAs have various biological functions, such as acting as microRNA or RBP sponges, regulating gene splicing and transcription, and translation into proteins [47]. Due to the cytoplasmic localization and the prediction of translation regulatory elements of circHEATR5B, we speculated that it had the potential to encode a novel protein, termed HEATR5B-881aa. We found that circHEATR5B-IRES could mediate 5′ cap-independent translation, and circHEATR5B-ORF could encode HEATR5B-881aa. Furthermore, HEATR5B-881aa overexpression mediated by circHEATR5B could be reversed by circHEATR5B-IRES mutation, indicating that HEATR5B-881aa is translated by circHEATR5B. Moreover, ZCRB1-mediated overexpression of HEATR5B-881aa further proves that the ZCRB1/circHEATR5B/HEATR5B-881aa pathway is active in GBM cells.

Studies have shown that proteins encoded by circRNAs can exert biological functions in tumor development [21, 22, 48, 49]. We wondered whether HEATR5B-881aa could affect GBM development. HEATR5B-881aa showed low expression in GBM and could exert suppressive effects on glycolysis and proliferation in GBM cells. To demonstrate that these effects were not induced by circHEATR5B itself, we mutated the start codon of circHEATR5B, which only interfered with its translation without altering its expression and structure. The loss of inhibitory effects indicates that these effects are induced by the encoded HEATR5B-881aa rather than by other potential functions of circHEATR5B itself, which is also proven by subsequent rescue experiments. In conclusion, it is by encoding HEATR5B-881aa that circHEATR5B suppresses glycolysis and proliferation in GBM cells.

To investigate the possible mechanisms by which HEATR5B-881aa regulated glycolysis and proliferation in GBM cells, we performed Co-IP assays coupled with mass spectrometry and selected JMJD5, which has been reported in breast cancer to bind and induce PKM2 dimerization, thereby regulating glucose metabolism [24]. PKM2 dimerization causes its enzymatic activity to decrease; hence, we detected PKM2 enzymatic activity in JMJD5-transfected GBM cells, and the results demonstrate that JMJD5 can equally promote PKM2 dimerization in GBM cells and thereby promote glycolysis and proliferation in GBM cells. Therefore, we conclude that the ZCRB1/circHEATR5B/HEATR5B-881aa/JMJD5 pathway regulates GBM glycolysis by targeting PKM2. In addition to its role as a glycolytic regulatory enzyme that promotes lactate fermentation in the cytoplasm, PKM2 can also translocate into the nucleus to enhance c-Myc and HIF-1α transcription and then increase glycolysis-related gene expression, including GLUT1, which regulates glucose uptake; LDHA, which regulates lactate production; MCT4, which regulates lactate transport [50–52]. Together, in GBM cells, these factors promote an increase in glucose uptake and lactate production, further causing the increase of extracellular H^+^ concentration measured by the Seahorse apparatus.

We found that HEATR5B-881aa reduced JMJD5 protein expression without influencing JMJD5 mRNA expression, and JMJD5 overexpression rescued glycolytic and proliferative inhibition induced by HEATR5B-881aa, indicating that HEATR5B-881aa inhibits glycolysis and proliferation in GBM cells by downregulating JMJD5 at the protein level. However, the mechanisms by which HEATR5B-881aa decreased JMJD5 protein levels remained unclear. We first identified a direct interaction between HEATR5B-881aa and JMJD5. Through HEATR5B-881aa domain analysis, we speculated that HEATR5B-881aa might regulate JMJD5 by phosphorylation modification. Further studies revealed that HEATR5B-881aa phosphorylated JMJD5 on S361 in vitro and in vivo. In addition, we found that JMJD5 expression was reduced as the S361 phosphorylation level increased, indicating that HEATR5B-881aa reduces JMJD5 by S361 phosphorylation. It has been reported that phosphorylation can affect protein stability [53]. In this study, JMJD5 phosphorylation significantly reduced JMJD5 stability, which was reversed by the proteasome inhibitor. This finding suggests that JMJD5 phosphorylation may mediate its degradation via the ubiquitin–proteasome pathway, which requires further investigation. In conclusion, HEATR5B-881aa reduces JMJD5 stability through S361 phosphorylation and thus downregulates its expression.

Finally, in vivo experiments showed that ZCRB1, circHEATR5B, or HEATR5B-881aa overexpression could inhibit the growth of subcutaneous GBM xenograft tumors and prolong the survival time of nude mice with orthotopic GBM xenograft tumors; furthermore, their combination had the strongest tumor-suppressive effect. These results further clarify the potential clinical value of ZCRB1, circHEATR5B, and HEATR5B-881aa in GBM.

## Conclusions

This study demonstrates for the first time that low expression of the RNA-binding protein ZCRB1 inhibits circHEATR5B formation, which further reduces expression of the encoded HEATR5B-881aa, resulting in the reduced phosphorylation and thus increased stability of JMJD5. High expression of JMJD5 induces PKM2 dimerization, thus promoting aerobic glycolysis and proliferation in GBM cells. This study reveals a novel mechanism of regulating aerobic glycolysis and proliferation in GBM cells through the ZCRB1/circHEATR5B/HEATR5B-881aa/JMJD5/PKM2 pathway, which can provide novel strategies and potential targets for GBM therapy.

## Supplementary Information

**Additional file 1: Figure S1.** Screening for ZCRB1, the mRNA expression of ZCRB1, and the transfection efficiency of ZCRB1 plasmids. **Figure S2.** Screening for circHEATR5B, the mRNA expression of HEATR5B, and the transfection efficiency of circHEATR5B plasmids. **Figure S3.** The effects of ZCRB1 on circHEATR5B stability and the binding prediction of ZCRB1 and circHEATR5B. **Figure S4.** The prediction of the potential encoding capacity of circHEATR5B and the expression of HEATR5B protein. **Figure S5.** Statistical analysis of western blot assays and ECAR parameter calculations. **Figure S6.** Screening for JMJD5 and the transfection efficiency of JMJD5 plasmids. **Figure S7.** JMJD5 phosphorylation induced by HEATR5B-881aa downregulated JMJD5 expression, which was reversed by MG132 treatment.

**Additional file 2: Table S1.** The primers for qRT–PCR assays. **Table S2.** The primers for RIP assays. **Table S3.** Top ten differentially expressed RBPs in GBM tissues screened by RNA-seq. **Table S4.** Top ten differentially expressed circRNAs in ZCRB1-upregulated GBM cells screened by circRNA microarray analysis. **Table S5.** The divergent and convergent primers of circHEATR5B and β-actin.

## Data Availability

The data that support the findings of this study are available from the corresponding author upon reasonable request.

## Abbreviations

5′-UTR: 5′-untranslated region
CBB: Coomassie brilliant blue
CCK-8: Cell Counting Kit-8
CHX: Cycloheximide
circHEATR5B: circRNA HEAT repeat containing 5B
circRNAs: circular RNAs
Co-IP: Co-immunoprecipitation
ECAR: Extracellular acidification rate
FISH: Fluorescence in situ hybridization
Fluc: Firefly luciferase
GBM: Glioblastoma multiforme
HEATR5B: HEAT repeat containing 5B
HGGTs: High-grade glioma tissues
IB: Immunoblotting
IDVs: Integrated density values
IF: Immunofluorescence
IP: Immunoprecipitation
IPTG: Isopropyl-β-D-1-thiogalactopyranoside
IRES: Internal ribosome entry site
JMJD5: Jumonji C-domain-containing 5
KDM8: Lysine (K) demethylase 8
LGGTs: Low-grade glioma tissues
n.s.: not significant
NBTs: Non-tumor brain tissues
NC: Negative control
NHA: Normal human astrocyte
ORF: Open reading frame
PKM2: Pyruvate kinase M2
qRT–PCR: quantitative reverse transcription PCR
RBPs: RNA-binding proteins
RIP: RNA Immunoprecipitation
Rluc: Renilla luciferase
RNA-seq: RNA sequencing
ROC: Receiver operating characteristic
SA: Splicing acceptor
SD: Splicing donor
WT: Wild-type
ZCRB1: Zinc finger CCHC-type and RNA-binding motif 1
