# Supplementary material for: A novel protein encoded by ZCRB1-induced circHEATR5B suppresses aerobic glycolysis of GBM through phosphorylation of JMJD5
Source: J Exp Clin Cancer Res. 2022 May 10;41:171. doi: 10.1186/s13046-022-02374-6 (PMC9086421; doi:10.1186/s13046-022-02374-6)
Supplement: Supplementary file 1 — Additional file 1: Figure S1. Screening for ZCRB1, the mRNA expression of ZCRB1, and the transfection efficiency of ZCRB1 plasmids. Figure S2. Screening for circHEATR5B, the mRNA expression of HEATR5B, and the transfection efficiency of circHEATR5B plasmids. Figure S3. The effects of ZCRB1 on circHEATR5B stability and the binding prediction of ZCRB1 and circHEATR5B. Figure S4. The prediction of the potential encoding capacity of circHEATR5B and the expression of HEATR5B protein. Figure S5. Statistical analysis of western blot assays and ECAR parameter calculations. Figure S6. Screening for JMJD5 and the transfection efficiency of JMJD5 plasmids. Figure S7. JMJD5 phosphorylation induced by HEATR5B-881aa downregulated JMJD5 expression, which was reversed by MG132 treatment. [file 13046_2022_2374_MOESM1_ESM.docx]

**
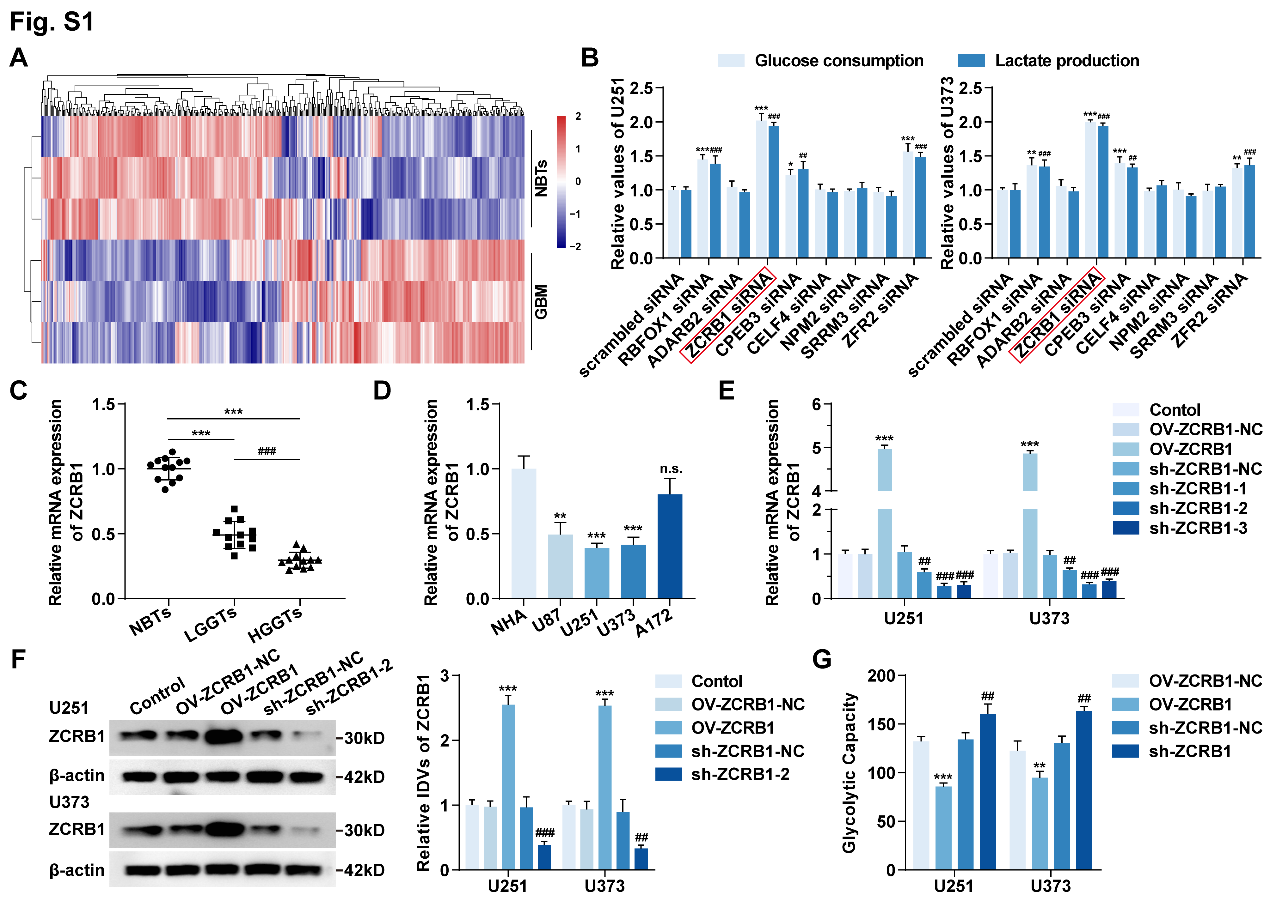
**

**Fig. S1 Screening for ZCRB1, the mRNA expression of ZCRB1, and the transfection efficiency of ZCRB1 plasmids.**

**(A)** Heatmap with hierarchical cluster analysis of differentially expressed genes between NBTs and GBM tissues by RNA-seq (*n* = 3, each group). *P* < 0.05, |log_2_FC| > 1. **(B)** The effects of RBPs knockdown on glucose consumption and lactate production in U251 and U373 cells. Data are presented as the mean ± SD (*n* = 3, each group). ^*^*P* < 0.05, ^**^*P* < 0.01, ^***^*P* < 0.001 vs. glucose consumption in scrambled siRNA group; ^##^*P* < 0.01, ^###^*P* < 0.001 vs. lactate production in scrambled siRNA group by one-way ANOVA. **(C)** The expression of ZCRB1 mRNA in the NBTs, LGGTs, and HGGTs groups. Data are presented as the mean ± SD (*n* = 12, each group). ^***^*P* < 0.001 vs. NBTs group; ^###^*P* < 0.001 vs. LGGTs group by one-way ANOVA. **(D)** The expression of ZCRB1 mRNA in NHA, U87, U251, U373, and A172 cells. Data are presented as the mean ± SD (*n* = 3, each group). n.s., ^**^*P* < 0.01, ^***^*P* < 0.001 vs. NHA group by one-way ANOVA. **(E and F)** Transfection efficiency of ZCRB1 plasmids in U251 and U373 cells detected by qRT–PCR (E) and western blot (F) assays. Data are presented as the mean ± SD (*n* = 3, each group). ^***^*P* < 0.001 vs. OV-ZCRB1-NC group; ^##^*P* < 0.01, ^###^*P* < 0.001 vs. sh-ZCRB1-NC group by one-way ANOVA. **(G)** Glycolytic capacity of ECAR in Fig. 1F. Data are presented as the mean ± SD (*n* = 3, each group). ^**^*P* < 0.01, ^***^*P* < 0.001 vs. OV-ZCRB1-NC group; ^##^*P* < 0.01 vs. sh-ZCRB1-NC group by one-way ANOVA.


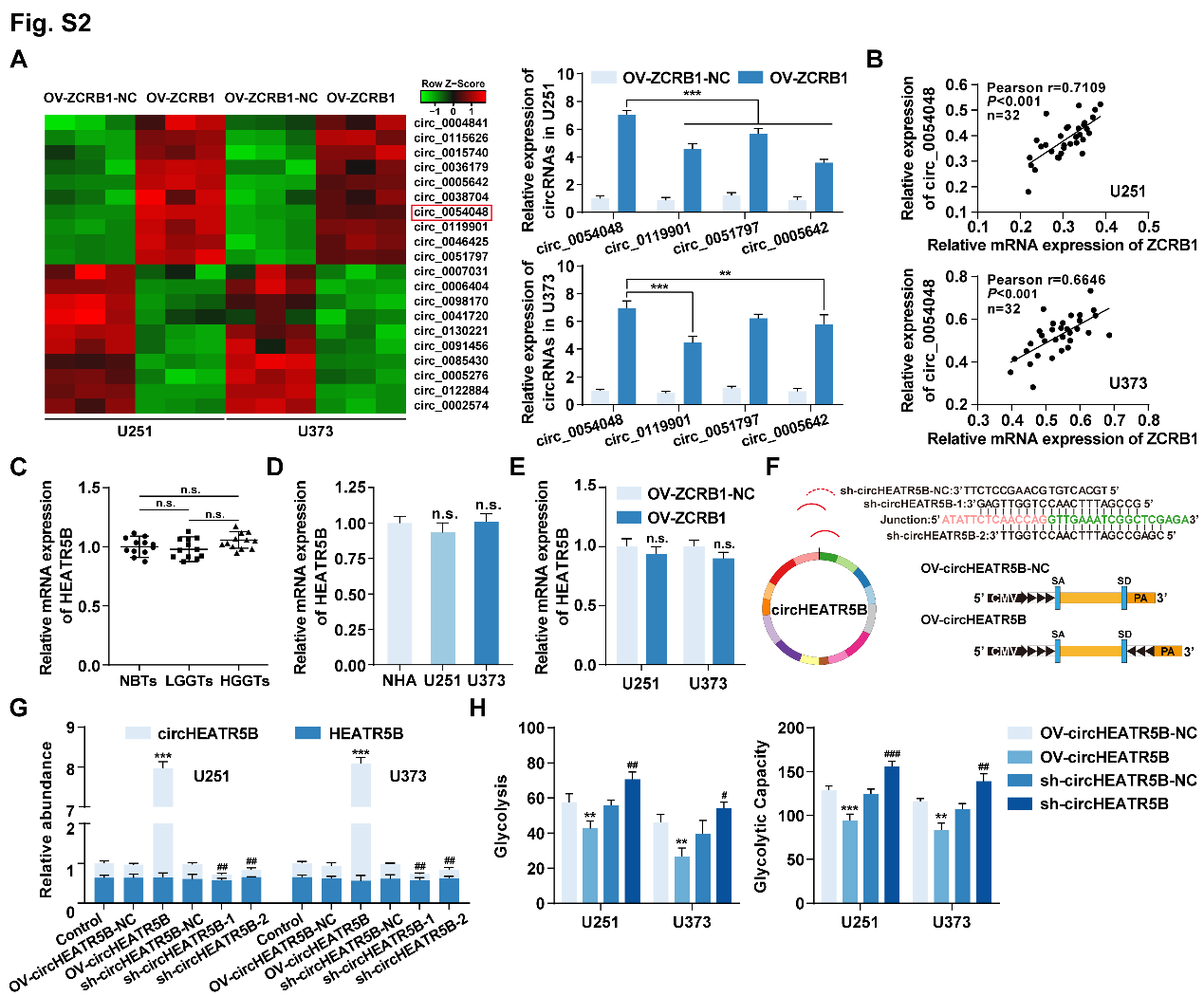


**Fig. S2 Screening for circHEATR5B, the mRNA expression of HEATR5B, and the transfection efficiency of circHEATR5B plasmids.**

**(A)** Left panel, expression profiles of differentially expressed circRNAs in ZCRB1-upregulated U251 and U373 cells according to circRNA microarray analysis. Right panel, the expression validation of four circRNA candidates screened by circRNA microarray profiles by qRT–PCR assays. Data are presented as the mean ± SD (*n* = 3, each group). ^**^*P* < 0.01, ^***^*P* < 0.001 vs. OV-ZCRB1 in circ_0054048 group by one-way ANOVA. **(B)** Linear correlation analysis of ZCRB1 mRNA and circ_0054048 expression in U251 and U373 cells. **(C)** The expression of HEATR5B mRNA in NBTs, LGGTs, and HGGTs groups. Data are presented as the mean ± SD (*n* = 12, each group). n.s. vs. NBTs group; n.s. vs. LGGTs group by one-way ANOVA. **(D)** The expression of HEATR5B mRNA in NHA, U251, and U373 cells. Data are presented as the mean ± SD (*n* = 3, each group). n.s. vs. NHA group by one-way ANOVA. **(E)** The expression of HEATR5B mRNA in response to ZCRB1 overexpression. Data are presented as the mean ± SD (*n* = 3, each group). n.s. vs. OV-ZCRB1-NC group by Student’s t-test. **(F)** Schematic illustration of circHEATR5B plasmid constructions. **(G)** Transfection efficiency of circHEATR5B plasmids in U251 and U373 cells. The expressions of circHEATR5B and HEATRB mRNA were detected simultaneously by qRT–PCR assays. Data are presented as the mean ± SD (*n* = 3, each group). ^***^*P* < 0.001 vs. circHEATR5B in OV-circHEATR5B-NC group; ^##^*P* < 0.01 vs. circHEATR5B in sh-circHEATR5B-NC group by one-way ANOVA. **(H)** Glycolysis and glycolytic capacity of ECAR in Fig. 2J. Data are presented as the mean ± SD (*n* = 3, each group). ^**^*P* < 0.01, ^***^*P* < 0.001 vs. OV-circHEATR5B-NC group; ^#^*P* < 0.05, ^##^*P* < 0.01, ^###^*P* < 0.001 vs. sh-circHEATR5B-NC group by one-way ANOVA.


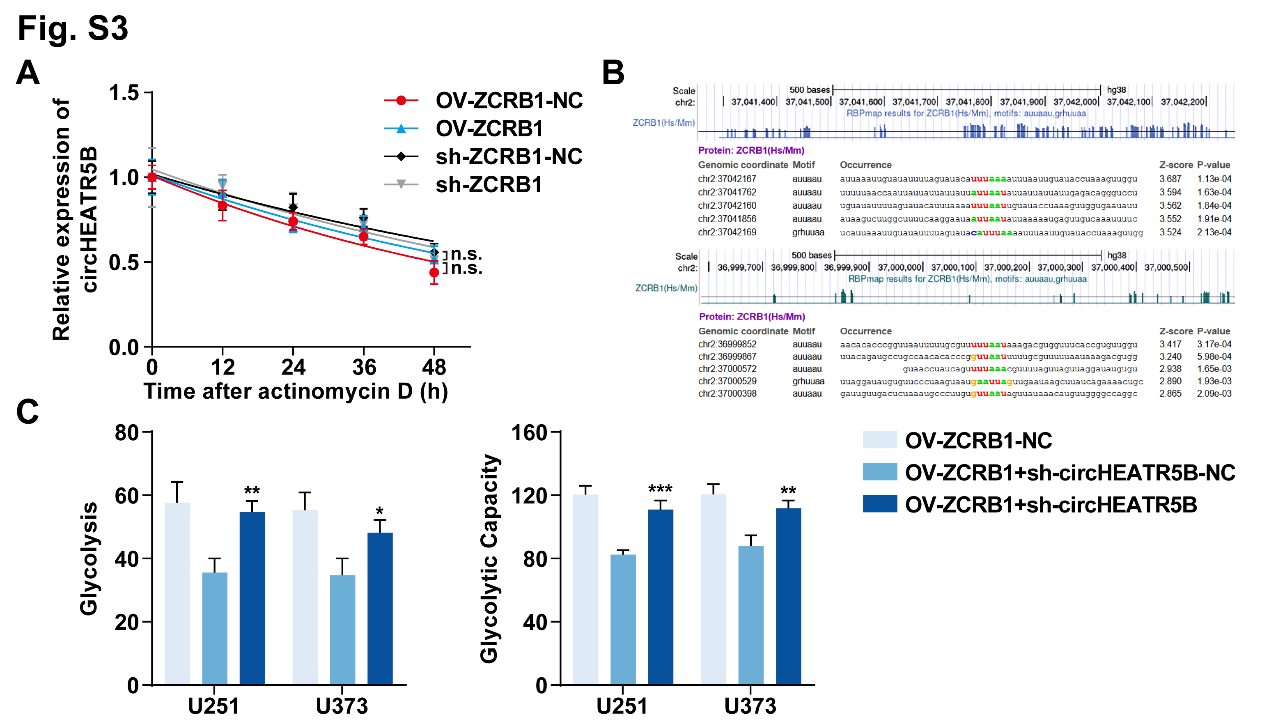


**Fig. S3** **The effects of ZCRB1 on circHEATR5B stability and the binding prediction of ZCRB1 and circHEATR5B.**

**(A)** The effects of ZCRB1 on circHEATR5B half-life were detected at the indicated time points by qRT–PCR assays in U251 cells after actinomycin D treatment. Data are presented as the mean ± SD (*n* = 3, each point). n.s. vs. OV-ZCRB1-NC group; n.s. vs. sh-ZCRB1-NC group by two-way ANOVA. **(B)** The binding sites of ZCRB1 to flanking sequences at both ends of circHEATR5B predicted by RBPmap server (http://rbpmap.technion.ac.il/). **(C)** Glycolysis and glycolytic capacity of ECAR in Fig. 3H. Data are presented as the mean ± SD (*n* = 3, each group). ^*^*P* < 0.05, ^**^*P* < 0.01, ^***^*P* < 0.001 vs. OV-ZCRB1+sh-circHEATR5B-NC group by one-way ANOVA.


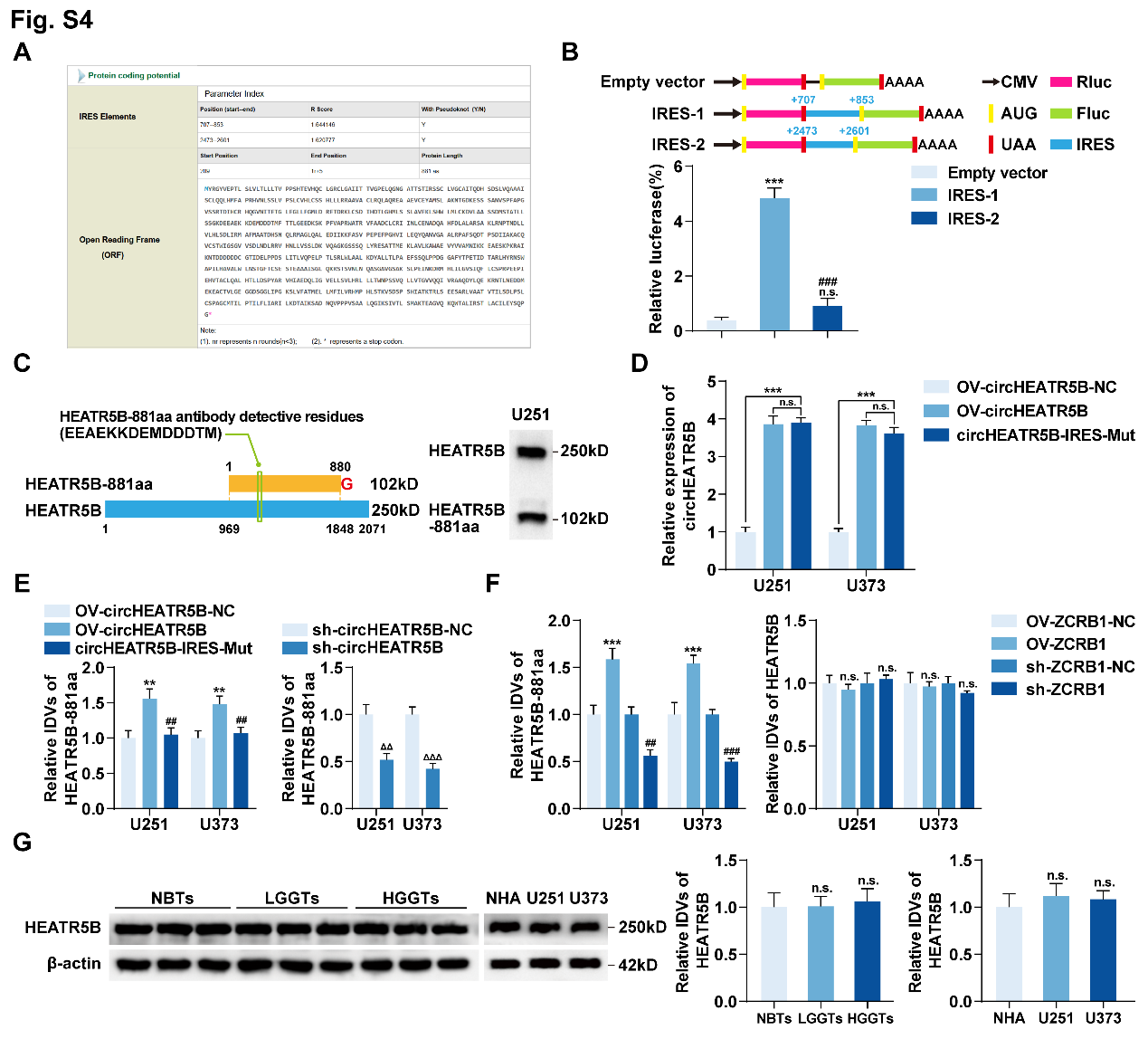


**Fig. S4 The prediction of the potential encoding capacity of circHEATR5B and the expression of HEATR5B protein.**

**(A)** The translation regulatory elements of circHEATR5B predicted by circRNADb database (http://reprod.njmu.edu.cn/circrnadb). **(B)** Upper panel, schematic of plasmids with IRES inserted between the Rluc stop codon and Fluc start codon. Lower panel, the luciferase activity of Fluc/Rluc was detected by dual-luciferase reporter assays. Data are presented as the mean ± SD (*n* = 3, each group). ^***^*P* < 0.001, n.s. vs. empty vector group; ^###^*P* < 0.001 vs. IRES-1 group by one-way ANOVA. **(C)** Schematic of the antigen design of the custom HEATR5B-881aa antibody and the antibody validation by western blot assay. **(D)** Transfection efficiency of circHEATR5B-IRES-Mut plasmid in U251 and U373 cells detected by qRT–PCR assays. Data are presented as the mean ± SD (*n* = 3, each group). ^***^*P* < 0.001 vs. OV-circHEATR5B-NC group; n.s. vs. OV-circHEATR5B group by one-way ANOVA. **(E)** Statistical analysis of the western blot assays in Fig. 4F. Data are presented as the mean ± SD (*n* = 3, each group). ^**^*P* < 0.01 vs. OV-circHEATR5B-NC group; ^##^*P* < 0.01 vs. OV-circHEATR5B group by one-way ANOVA. ^ΔΔ^*P* < 0.01, ^ΔΔΔ^*P* < 0.001 vs. sh-circHEATR5B-NC group by Student’s t-test. **(F)** Statistical analysis of the western blot assays in Fig. 4G. Data are presented as the mean ± SD (*n* = 3, each group). ^***^*P* < 0.001, n.s. vs. OV-ZCRB1-NC group; ^##^*P* < 0.01, ^###^*P* < 0.001, n.s. vs. sh-ZCRB1-NC group by one-way ANOVA. **(G)** The expression of HEATR5B protein in NBTs, LGGTs, and HGGTs groups. Data are presented as the mean ± SD (*n* = 9, each group). n.s. vs. NBTs group; n.s. vs. LGGTs group by one-way ANOVA. The expression of HEATR5B protein in NHA, U251, and U373 cells. Data are presented as the mean ± SD (*n* = 3, each group). n.s. vs. NHA group by one-way ANOVA.


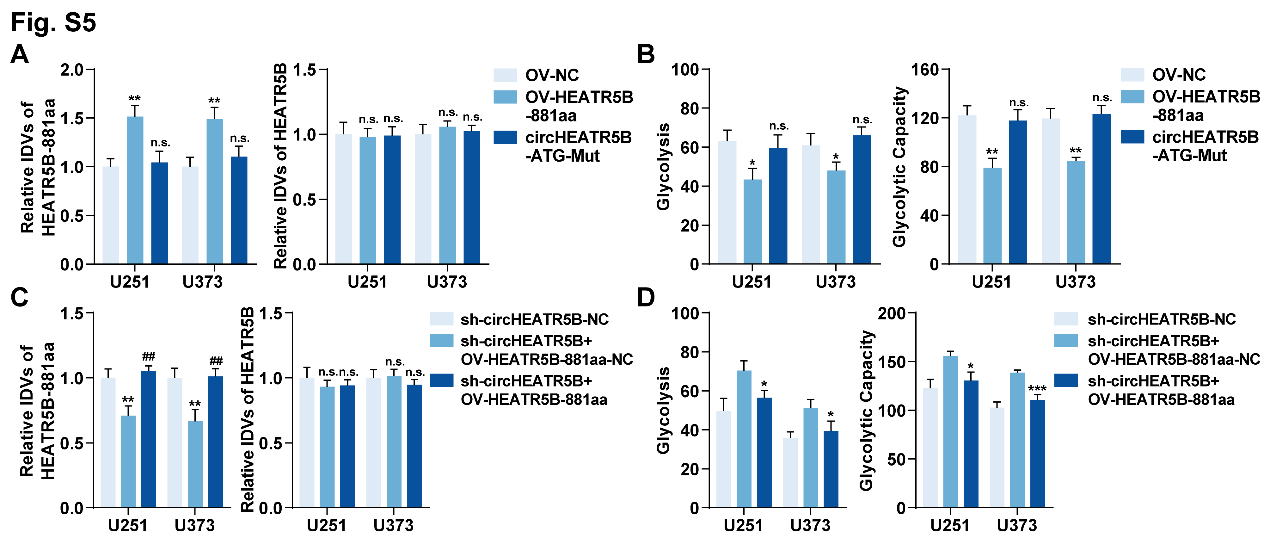


**Fig. S5 Statistical analysis of western blot assays** **and ECAR parameter calculations.**

**(A)** Statistical analysis of the western blot assays in Fig. 5A. Data are presented as the mean ± SD (*n* = 3, each group). ^**^*P* < 0.01, n.s. vs. OV-NC group by one-way ANOVA. **(B)** Glycolysis and glycolytic capacity of ECAR in Fig. 5D. Data are presented as the mean ± SD (*n* = 3, each group). ^*^*P* < 0.05, ^**^*P* < 0.01, n.s. vs. OV-NC group by one-way ANOVA. **(C)** Statistical analysis of the western blot assays in Fig. 5F. Data are presented as the mean ± SD (*n* = 3, each group). ^**^*P* < 0.01, n.s. vs. sh-circHEATR5B-NC group; ^##^*P* < 0.01, n.s. vs. sh-circHEATR5B+OV-HEATR5B-881aa-NC group by one-way ANOVA. **(D)** Glycolysis and glycolytic capacity of ECAR in Fig. 5I. Data are presented as the mean ± SD (*n* = 3, each group). ^*^*P* < 0.05, ^***^*P* < 0.001 vs. sh-circHEATR5B+OV-HEATR5B-881aa-NC group by one-way ANOVA.


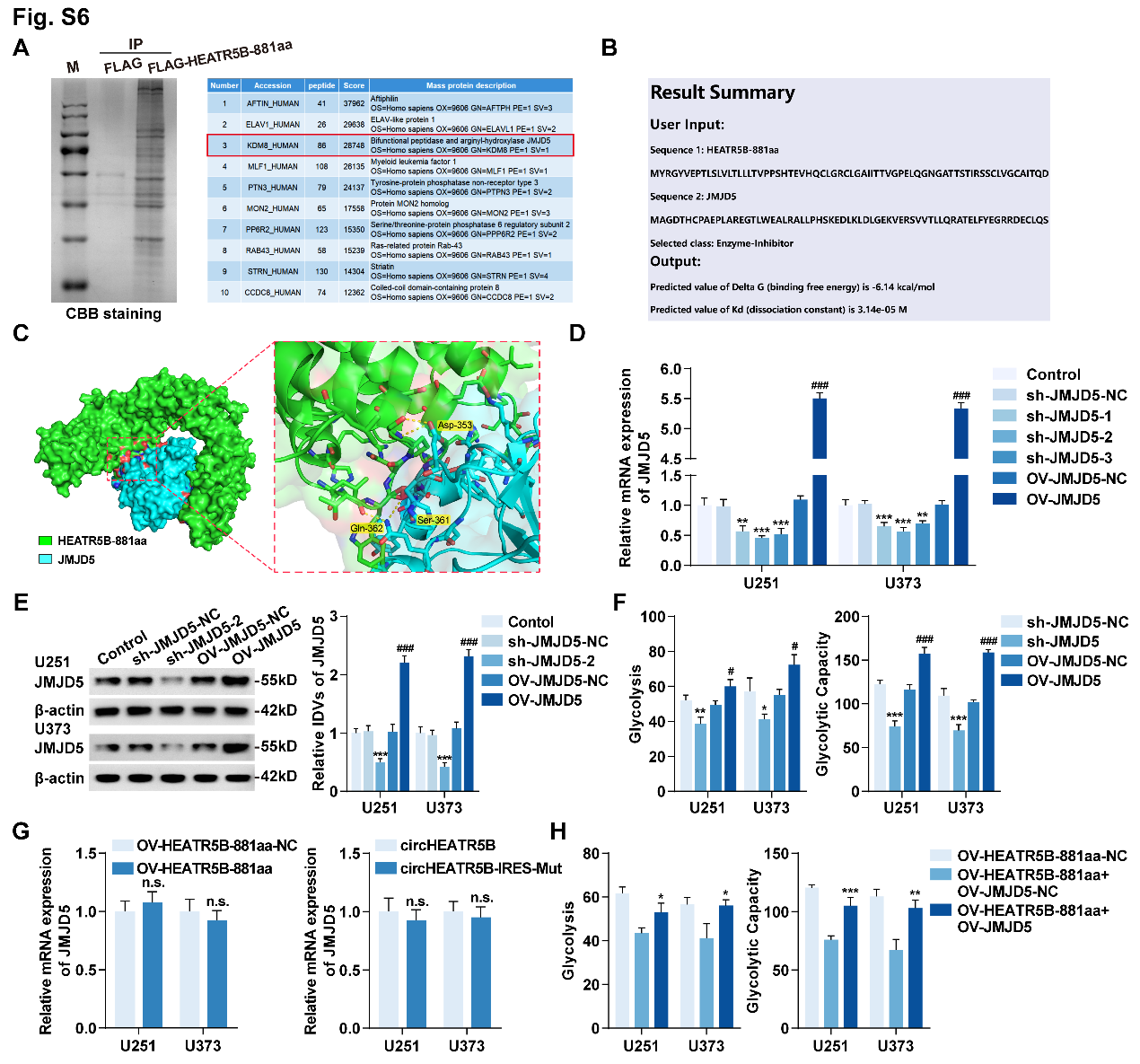


**Fig. S6 Screening for JMJD5 and the transfection efficiency of JMJD5 plasmids.**

**(A)** Left panel, Coomassie brilliant blue (CBB) staining of precipitates immunoprecipitated by FLAG tag antibodies from the cytoplasmic fractions of U251 cells transfected with FLAG-HEATR5B-881aa or empty plasmid. Right panel, HEATR5B-881aa–interacting proteins screened by mass spectrometry analysis of the precipitates, and JMJD5 (also named KDM8) was marked with a red frame. **(B)** The binding affinity of HEATR5B-881aa and JMJD5 predicted by the PPA-Pred2 server (http://www.iitm.ac.in/bioinfo/PPA_Pred/). **(C)** Left panel, docking complex by the ZDOCK server (http://zdock.umassmed.edu) exhibiting the surface interaction between HEATR5B-881aa (green) and JMJD5 (cyan, Protein Data Bank ID: 6F4M). Right panel, an enlarged view of the interaction interface of HEATR5B-881aa–JMJD5 complex. Hydrogen bonds are shown in yellow dotted lines. **(D and E)** Transfection efficiency of JMJD5 plasmids in U251 and U373 cells by qRT–PCR (D) and western blot (E) assays. Data are presented as the mean ± SD (*n* = 3, each group). ^**^*P* < 0.01, ^***^*P* < 0.001 vs. sh-JMJD5-NC group; ^###^*P* < 0.001 vs. OV-JMJD5-NC group by one-way ANOVA. **(F)** Glycolysis and glycolytic capacity of ECAR in Fig. 6G. Data are presented as the mean ± SD (*n* = 3, each group). ^*^*P* < 0.05, ^**^*P* < 0.01, ^***^*P* < 0.001 vs. sh-JMJD5-NC group; ^#^*P* < 0.05, ^###^*P* < 0.001 vs. OV-JMJD5-NC group by one-way ANOVA. **(G)** The expression of JMJD5 mRNA in response to HEATR5B-881aa detected by qRT–PCR assays in U251and U373 cells. Data are presented as the mean ± SD (*n* = 3, each group). n.s. vs. OV-HEATR5B-881aa-NC group; n.s. vs. circHEATR5B group by Student’s t-test. **(H)** Glycolysis and glycolytic capacity of ECAR in Fig. 6M. Data are presented as the mean±SD (n=3, each group). ^*^*P* < 0.05, ^**^*P* < 0.01, ^***^*P* < 0.001 vs. OV-HEATR5B-881aa+OV-JMJD5-NC group by one-way ANOVA.


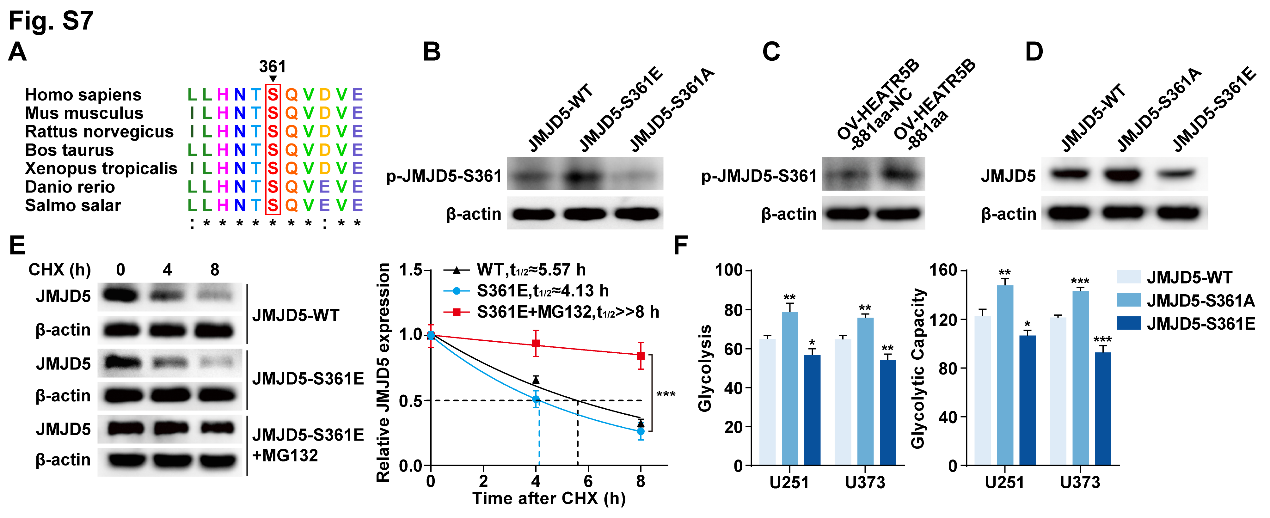


**Fig. S7 JMJD5 phosphorylation induced by HEATR5B-881aa downregulated JMJD5 expression, which was reversed by MG132 treatment.**

**(A)** Conservation analysis of the phosphorylation site S361 in different species. **(B)** The S361 phosphorylation levels were detected by the custom p-JMJD5-S361 antibody in U251 cells transfected with JMJD5-WT, JMJD5-S361E, or JMJD5-S361A plasmid. **(C)** The S361 phosphorylation levels were detected in HEATR5B-881aa–upregulated U251 cells. **(D)** The expression of JMJD5 was detected in JMJD5-downregulated U251 cells transfected with JMJD5-WT, JMJD5-S361A, or JMJD5-S361E plasmid. **(E)** The half-life of JMJD5 was measured after CHX treatment with or without proteasome inhibitor MG132. Data are presented as the mean ± SD (*n* = 3, each group). ^***^*P* < 0.001 vs. S361E group by two-way ANOVA. **(F)** Glycolysis and glycolytic capacity of ECAR in Fig. 7L. Data are presented as the mean ± SD (*n* = 3, each group). ^*^*P* < 0.05, ^**^*P* < 0.01, ^***^*P* < 0.001 vs. JMJD5-WT group by one-way ANOVA.
